# Supplementary material for: Interactions between Fermi polarons in monolayer WS2
Source: Nat Commun. 2022 Oct 18;13:6164. doi: 10.1038/s41467-022-33811-x (PMC9579159; doi:10.1038/s41467-022-33811-x)
Supplement: Supplementary file 1 — Supplementary Information [file 41467_2022_33811_MOESM1_ESM.pdf]

Supplementary Information: Interactions between Fermi polarons in monolayer WS_2

Jack B. Muir,[1,2] Jesper Levinsen,[3,2] Stuart K. Earl,[1,2] Mitchell A. Conway,[1,2] Jared H. Cole,[4,2] Matthias Wurdack,[5,2] Rishabh Mishra,[1,2] David J. Ing,[4] Eliezer Estrecho,[5,2] Yuerui Lu,[6,7] Dmitry K. Efimkin,[3,2] Jonathan O. Tollerud,[1] Elena A. Ostrovskaya,[5,2] Meera M. Parish,[3,2] and Jeffrey A. Davis[1,2]

[1] *Optical Sciences Centre, Swinburne University of Technology, Victoria 3122, Australia*

[2] *ARC Centre of Excellence in Future Low-Energy Electronics Technologies*

[3] *School of Physics and Astronomy, Monash University, Victoria 3800, Australia*

[4] *Chemical and Quantum Physics, School of Science, RMIT University, Melbourne, VIC 3001, Australia*

[5] *Research School of Physics, The Australian National University, Canberra, ACT 2601, Australia*

[6] *School of Engineering, College of Engineering and Computer Science, The Australian National University, Canberra, ACT 2601, Australia*

[7] *ARC Centre of Excellence for Quantum Computation and Communication Technology, the Australian National University, Canberra, ACT, 2601 Australia*

(Dated: September 29, 2022)

SUPPLEMENTARY NOTE 1: SAMPLE CHARACTERIZATION

A. Optical properties

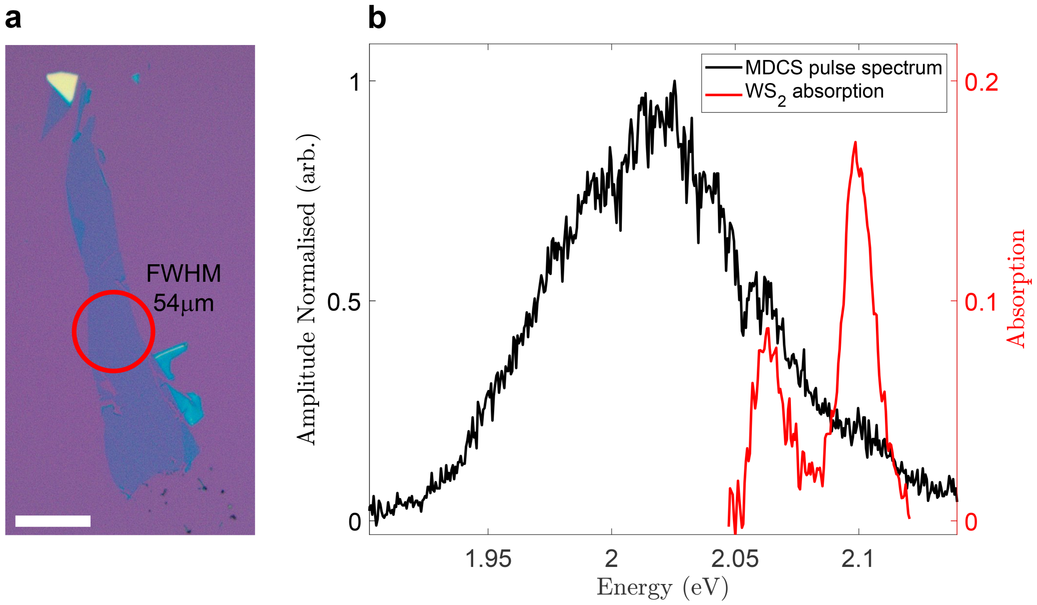

Supplementary Figure 1 | Optical properties of the exfoliated monolayer WS_2 on Si/SiO_2. a, Bright field optical image of the sample. The red circle indicates the size and location of the laser spots used in the MDCS experiments. The sample is slightly overfilled by the laser spots, but because there is no response from the substrate this doesn't affect the measurements. The scalebar is 50 µm. **b**, Absorbance of the sample (red) at T=6 K, and MDCS pulse spectrum (black).

To quantify the absorption of the monolayer, reflected spectra of the local oscillator (LO) were collected from the WS_2 flake and the adjacent substrate at 6 K. The spectra were normalised, to account for slight variations in the laser spectrum, and the difference between the spectra was found and taken to approximate the absorption of the flake. While this measurement is simplistic, after accounting for the narrowing and blueshift of the excitonic resonances it agrees well with previous, more rigorous calculations performed using the transfer matrix method performed at room temperature [1].

A thin film model was also applied to calculate the reduction in the incident laser intensity at the location of the monolayer on the layered SiO_2/Si substrate (300 nm thermal oxide) due to interference effects [2]. This found that due to the interference of the incoming and reflected waves from the substrate the intensity at the location of the WS_2 monolayer was reduced to around 41% of the incident intensity at the peak of the A exciton. This reduction was taken into account when calculating the exciton density in the WS_2 monolayer.

B. Estimating electron doping

For the MDCs measurements we have laser spot sizes on the order of 50 μm diameter, which means that to minimise scatter we need a clear monolayer area of similar size. This makes it difficult to obtain a precise measure of electron density, as having contacts on the sample becomes problematic. We took two different approaches to estimate the electron density.

First, we took gate voltage dependent measurements on another identically-prepared sample taken from the same bulk crystal. There are problems with such measurements on un-encapsulated monolayer WS_2 due to the adsorption and de-adsorption of environmental molecules which effectively change the Fermi level. Differences then appear depending on whether the sample is in vacuum or in atmosphere, and over time when an applied gate voltage increases the electron density, as discussed in detail below. With these challenges, it was only possible to place an upper limit on the difference in Fermi energy from atmospheric to vacuum conditions of 15 meV. If we assume that the atmospheric conditions effectively quench any intrinsic doping, then this also places an upper limit on the Fermi energy for the MDCS measurements, which were conducted in vacuum.

The second approach was to compare the ratio of the exciton and trion peaks in the photoluminescence and low-temperature absorption measurements with literature values, since this ratio is strongly dependent on electron density. In our absorption measurements the exciton peak amplitude is ~ 5 times larger than the trion peak. There are limited measurements of absorption spectra as a function of doping density, but theory work looking at MoS_2 showed that for a Fermi energy of 4.9 meV an exciton absorption five times the trion absorption was reported [3]. This ratio is similar to what we see in our WS_2 monolayer under vacuum at 6K, and can provide a rough estimate. However, there has been no experimental confirmation, and the different conduction band ordering in WS_2 may change things significantly. For room temperature PL measurements, reported electron densities typically range from $< 1 \times 10^{11}$ to $2 \times 10^{12}\,\mathrm{cm}^{-2}$ but without a consistent quantitative trend for the relative peak amplitudes. Perhaps the most reliable report, where they have used two independent methods to determine the doping density, shows that even for electron densities less than $1 \times 10^{11}\,\mathrm{cm}^{-2}$ the trion peaks remain stronger than the exciton peak in photoluminescence measurements [4]. In the room temperature PL measurements in Supplementary Fig. 2b,

the PL from the trion is less than two times larger than the exciton, and consistent with a doping density $\sim 1 \times 10^{11}\,\mathrm{cm}^{-2}$. We used this value as a starting point for our modelling, but note that there remains significant uncertainty. In the modelling we varied this doping density and found that the best match with the experiment was obtained with a value of $1 \times 10^{11}\,\mathrm{cm}^{-2}$, and results consistent with the experiments were obtained for values up to $3 \times 10^{11}\,\mathrm{cm}^{-2}$.

C. Vacuum effect and gate voltage dependent measurements in monolayer WS_2

To investigate the effect of vacuum on the optical response of the WS_2 used in this study, we performed photoluminescence (PL) measurements under non-resonant excitation with a Nd:YAG continuous wave (cw) laser source (λ_{ex}=532 nm) in both air and vacuum at ambient temperature. The respective spectra were fitted with three Voigt profiles for neutral excitons (X), charged excitons (X^-) and localized excitons (X^L) with the energies $E_X \approx 2.02\,\mathrm{eV}$, $E_{X^-} \approx 1.99\,\mathrm{eV}$ and $E_{X^L} \approx 1.93\,\mathrm{eV}$, which is in agreement with previous reports [5, 6]. In air (Supplementary Fig. 2a), the PL stems mostly from neutral exciton emission with the exciton spectral weight of $w_X \approx 0.91 \pm 0.05$. However, the asymmetric line shape points to a non-negligible contribution of charged and localized excitons, which are represented by the fitted red and green shaded peaks in Supplementary Fig. 2, respectively. Localized excitons can form in a WS_2 crystal at certain defects [7] and charged excitons form when the monolayer is doped [8]. In addition, defects can increase the level of doping and enhance the formation of charged and localized excitons [9]. Physisorption gating [10] by oxygen and water molecules can compensate this defect-induced doping, which makes the resulting effects on the PL less significant in air. However, when placing the monolayer into vacuum ($p \approx 1 \times 10^{-6}$ mbar), the physisorbed molecules are released from the sample surface, and the contributions of the charged and localized excitons to the PL spectrum increase dramatically (Supplementary Fig. 2b) with the spectral weights of $w_{X^-} \approx 0.56 \pm 0.10$ and $w_{X^L} \approx 0.18 \pm 0.08$, respectively.

To measure the difference between the Fermi levels in air and vacuum, we emulated the vacuum effect (i.e. uncompensated defect-induced doping) by contacting a monolayer WS_2 sample on top of a highly doped Si/SiO_2 substrate ($t_{SiO_2} \approx 300$ nm, $\epsilon_{SiO_2} \approx 3.9$ [11]) acting as a backgate, as illustrated schematically in Supplementary Fig. 2c, which provides the

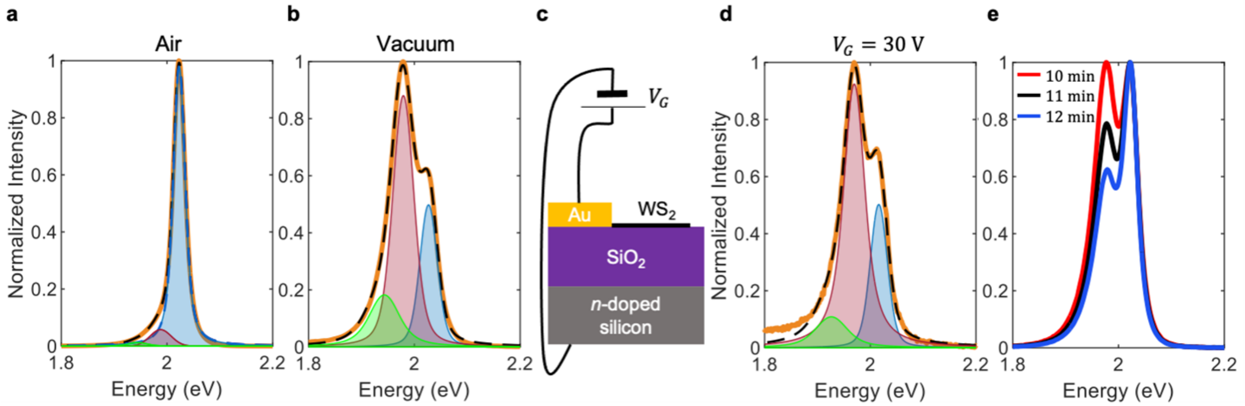

Supplementary Figure 2 | Physisorption gating measurements. **a**,**b**, Photoluminescence (PL) spectrum of a WS_2 monolayer (a) in air and (b) in vacuum (orange solid lines). **c**, Schematic illustration of a backgated WS_2 monolayer. **d**, PL spectrum of a gated WS_2 monolayer in air at V_G=30 V. The PL spectra in panels (a,b,d) were fitted with three Voigt profiles (black dashed lines) for the neutral (blue shaded), charged (red shaded) and localized excitons (green shaded). **e**, PL spectra in air at a gate voltage of 60 V measured 10 (red), 11 (black), and 12 (blue) minutes after the voltage was applied.

ability to tune the Fermi level across the band gap. Supplementary Figure 2d shows the PL spectrum at $V_G = 30\,\mathrm{V}$ in air, with the spectral weight of the charged excitons, $w_{X^-} \approx 0.66\pm0.04$, similar to their spectral weight in vacuum. The approximate difference between the Fermi levels in vacuum and air can be calculated as [12]:

$$\Delta E_F = \frac{\pi \hbar^2 \epsilon_{SiO_2} \epsilon_0}{t_{SiO_2} e^2 m^*} V_G \approx 15\mathrm{meV} \qquad (1)$$

where the effective electron mass in the conduction band is $m^* = 0.35 \cdot m_e$ [13].

Despite the significant redistribution of the spectral weight towards a charged exciton seen in a gated sample (Supplementary Fig. 2d), we find that the spectral weight of the neutral excitons increases over time once a fixed voltage is applied (Supplementary Fig. 2e). A feasible explanation is that, at higher voltages, more water and air molecules are able to bind to the monolayer surface, which leads to increasing physisorption gating that counteracts the external gating. Hence, the Fermi energy difference between the samples in air and in vacuum extracted above gives an upper limit of the actual value.

SUPPLEMENTARY NOTE 2: FURTHER EXPERIMENTAL DETAILS

A. Experimental geometry and pulse sequences

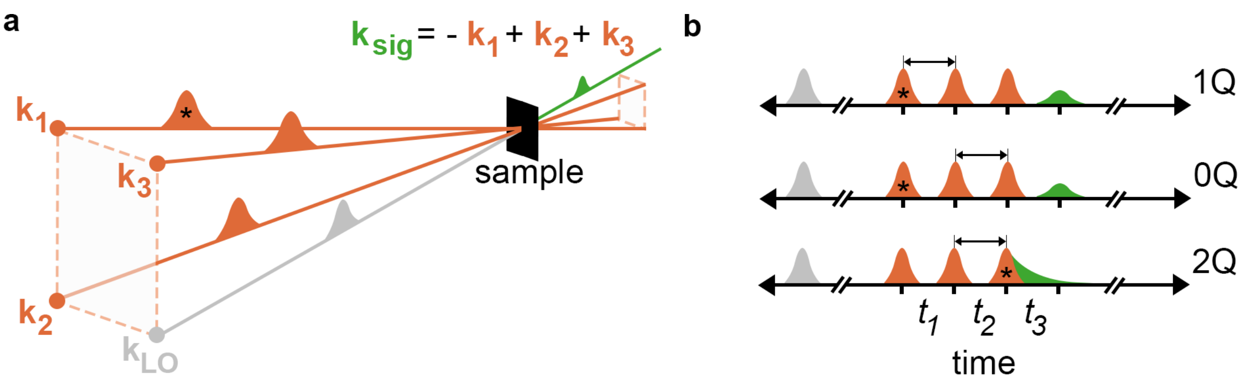

Supplementary Figure 3 | Experimental laser and pulse geometry. **a**, Four-beam box geometry used in our MDCS experiments, with the wavevector of each pulse labelled. The four-wave mixing signal emitted in the direction given by -$\mathbf{k}_1$ + $\mathbf{k}_2$ + $\mathbf{k}_3$ overlaps with the local oscillator on the fourth corner of the box. The negative contribution from $\mathbf{k}_1$ arises from a complex conjugate in the equations, which also impacts the time evolution of the phase of the signal. The $\mathbf{k}_1$ pulse is thus marked with (*) for reference. For polarization control $\mathbf{k}_1$ and $\mathbf{k}_3$ are σ^+, while $\mathbf{k}_2$ and $\mathbf{k}_{\mathrm{LO}}$ can either be σ^+ or σ^- corresponding to co-circular and cross-circular polarization schemes respectively. **b**, Different pulse orderings and delays being scanned for the different types of MDCS measurements; one-quantum (1Q), zero-quantum (0Q), and two-quantum (2Q). The arrow above the pulses indicates the time period that is scanned.

In the main text and Supplementary Information we report results from three types of MDCS measurements, referred to as one-quantum (1Q), zero-quantum (0Q), and two-quantum (2Q). Experimentally the difference between each of these measurements is simply related to the pulse ordering and which delay is scanned, as shown in Supplementary Fig. 3b. This is also impacted by the geometry of the experiments and which four-wave mixing signal is detected. Our experimental geometry is depicted in Supplementary Fig. 3a, which shows the measured signal is emitted in the direction given by -$\mathbf{k}_1$ + $\mathbf{k}_2$ + $\mathbf{k}_3$. The negative contribution from $\mathbf{k}_1$ arises from a complex conjugate in the equations, which also impacts the time evolution of the phase of the signal. For 1Q measurements the delay between the first two pulses is scanned. In this time period the system evolves in a coherent superposition between ground and excited states. In the pulse ordering shown, with the $\mathbf{k}_1$ pulse first and referred to a rephasing pulse ordering, any decrease of the macroscopic coherence as a result of inhomogeneous broadening can be “rephased” in the third time period due to the different direction of the phase evolution. This rephasing leads to a photon echo, and emission of the signal at a time $t_3 = t_1$. It is this rephasing and photon echo that causes the narrowing of the 2D peaks along the anti-diagonal direction and reveals the homogeneous linewidth, such

as in Fig. 2. In the case where the $\mathbf{k}_2$ pulse arrives first, the phase evolution in the first and third time periods is in the same direction, and there is no rephasing. This is referred to as the non-rephasing pulse ordering. For 0Q 2D spectra the pulse ordering doesn't change, but instead of scanning t_1, the delay between the second and third pulses, t_2 is scanned. For the 2Q scans shown below, the pulse ordering is changed so that the $\mathbf{k}_1$ pulse arrives last. In this case the first two pulses generate a coherence between the ground and doubly excited state, and its phase evolution is measured by scanning the t_2 delay.

B. Intensity Dependence

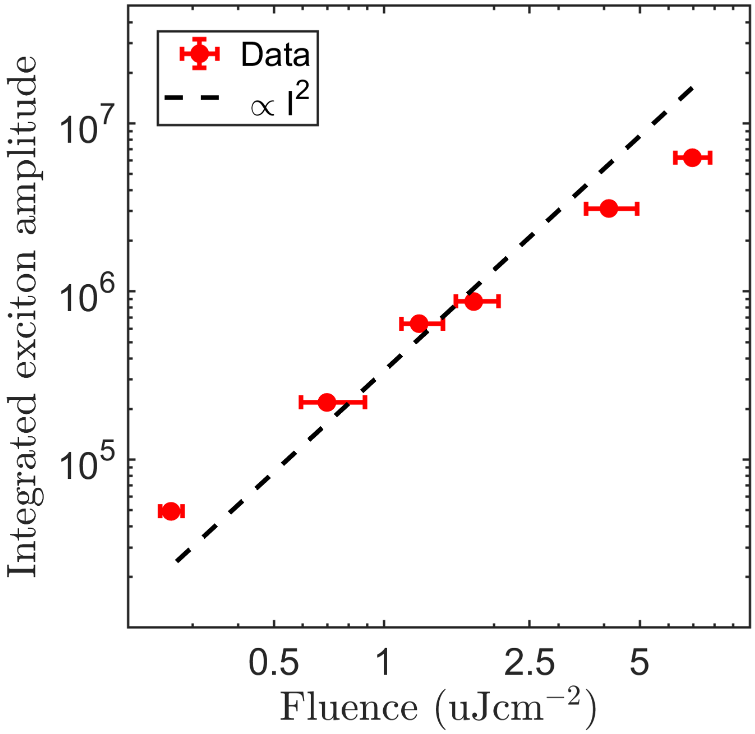

Supplementary Figure 4 | Power dependent measurement of the exciton. Integrated exciton signal from co-linear polarized 1Q spectra, and pulse fluence per beam calculated from laser specifications. Both axes are on a log scale to show data points follow a square dependence (dashed line). Measurements for this work were taken at ~0.9 µJ cm^{-2}. The fluence error bars are calculated from small differences in power and uncertainty in the spot size measurements for the three excitation pulses.

To ensure that the MDCS measurements were conducted at an intensity at which the third-order susceptibility dominates (and contributions from higher order effects can be ignored), we measured the signal amplitude as a function of pulse fluence. The electric field amplitude of the four-wave mixing signal (proportional to the third-order response of the sample) scales as the product of the electric field amplitude, E, in each excitation pulse, that

is, as E^3. The signal is detected interferometrically by combining with the local oscillator (LO). The amplitude measured is therefore proportional to the product of the signal and LO electric fields. Thus, in the third-order regime, the measured signal amplitude should scale as E^4, or intensity squared (I^2) (which is proportional to fluence squared). A sample response which deviates from this dependence is therefore indicative of higher order contributions. Supplementary Figure 4 confirms our data follows a square dependence (dashed line) for fluences up to at least $8\,\mu J\,cm^{-2}$ per beam, indicating an excitation regime where third-order effects dominate and higher order effects are minimised [14].

To obtain an estimate of the exciton density we assume one absorbed photon creates one exciton. The laser photon density can be calculated by dividing the fluence per pulse ($0.9\,\mu J\,cm^{-2}$) by the photon energy (which we take as the centre of the MDCS spectrum, 2.02 eV). The MDCS pulse spectrum is corrected to 41% of the incident intensity, and multiplied by the WS_2 absorption from Supplementary Fig. 1b. The ratio results in $\sim 0.5\%$ absorbed photons. Lastly, multiplying 0.5% by the calculated photon density yields the density of absorbed photons. From this, we estimate our exciton density is $1.3 \times 10^{10}\,cm^{-2}$, which is roughly an order of magnitude smaller than our estimated total electron density of $1 \times 10^{11}\,cm^{-2}$.

SUPPLEMENTARY NOTE 3: FULL 2D SPECTRA

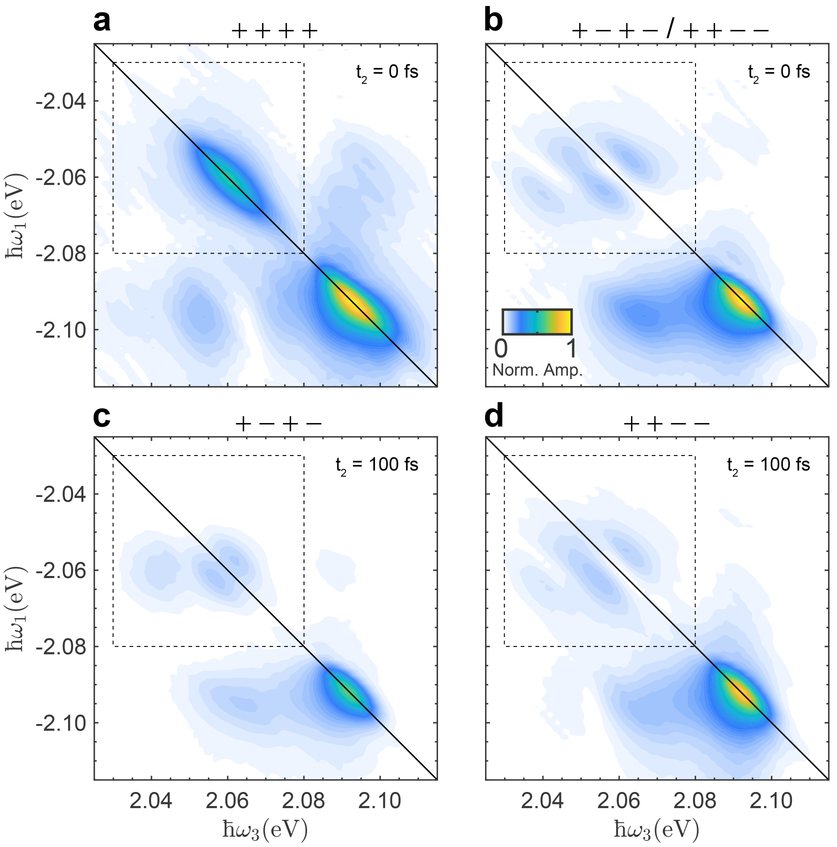

Supplementary Figure 5 | Full 1Q MDCS spectra for different polarization schemes. a-d, The 2D spectra over the full signal range for polarization schemes; (a) co-circular, (b) cross-circular, (c) cross-circular with t_2=100 fs: $\sigma^+\sigma^-\sigma^+\sigma^-$, (d) cross-circular with t_2=100 fs: $\sigma^+\sigma^+\sigma^-\sigma^-$. The colour scales in (a) and (b) are normalised to their respective exciton peak amplitudes, while (c) and (d) are normalised to the exciton peak in (b). The dotted box indicates the axes range used in the main text. In the $\sigma^+\sigma^-\sigma^+\sigma^-$ ordering the phase evolution of the S-T coherent superposition shifts the centre of these cross-peaks in towards the diagonal. The roughly equal amplitudes of the attractive polaron peaks evident in (c) and (d) confirm that both pathways contribute equally to the cross-peaks discussed in the main text.

In the main text, we focus our investigation on the substructure of the attractive polaron region, leaving the repulsive polaron region out of discussion. In Supplementary Fig. 5a,b we show the full spectra for the two polarization schemes in the main text. These plots are dominated by the repulsive polaron diagonal peak, which is two times larger than the attractive polaron peaks in the co-circular, and four times larger in the cross-circular.. Taking into account the laser spectrum, which has intensity at the attractive polaron energy more than double what it is at the repulsive polaron energy, the nonlinear response of the attractive polarons is $\sim 10 - 20$ times weaker than for the attractive polaron. Cross-peaks between attractive and repulsive polarons can also be identified, as well as a peak due to biexcitons [15]. As discussed in the main text, the cross-circular polarization data incor-

porates two pathways as a result of having $t_2 = 0$: $\sigma^+\sigma^-\sigma^+\sigma^-$ and $\sigma^+\sigma^+\sigma^-\sigma^-$; that is, the order of the second and third light matter interactions can be swapped. In Supplementary Fig. 5c and d, these pathways are separated by setting t_2=100 fs. On first inspection these 2D spectra show that the two different pathways contribute approximately equally, as predicted by the modelling. They are not identical, however, because in the $\sigma^+\sigma^-\sigma^+\sigma^-$ scheme the systems evolves in t_2 as a coherent superposition of S and T polarons, and when t_2=100 fs the phase of these peaks has changed, which, when added to the broad background signal, causes the centre of the cross-peaks to move in towards the diagonal.

SUPPLEMENTARY NOTE 4: QUANTUM MECHANICAL PATHWAY ANALYSIS

The measurements (Supplementary Fig. 2 in the main text) show that the co-circularly polarized 2D spectrum only shows diagonal attractive polaron peaks, while the cross-circularly polarized 2D spectrum only had cross-peaks. To understand the nature of these peaks, we list every possible (30 total) quantum mechanical (QM) pathway in our co-circular and cross-circular polarization schemes. Supplementary Figure 6 summarises the QM pathways with Feynman-Liouville diagrams [16], where the listed $|\rangle$ and $\langle|$ slot into the green (or orange) boxes, and their associated shapes indicate positions on the 2D spectra.

In the absence of interactions, these pathways will all cancel and there would be no signal. Specifically, the excited state absorption (ESA) pathways would cancel with the respective stimulated emission (SE) and/or ground state bleach (GSB) pathway. In the case of the co-circular polarization (diagonal peaks), there is only one ESA pathway, but both SE and GSB pathways. Because the ESA pathway requires the creation of a second indistinguishable polaron, the transition dipole moment of that transition is a factor of $\sqrt{2}$ larger, leading to a signal that is twice the amplitude, which can thus cancel with the SE and GSB pathways. In general, the presence of interactions leads to imperfect cancellation of these pathways, and the appearance of peaks in the 2D spectra.

This statement that there is no signal in the absence of interactions, even for diagonal peaks, may seem counter-intuitive to those accustomed to thinking of these experiments in the context of two-level systems (or few-level systems), where simply the presence of a bright state generates a peak on the diagonal. In the two-level system, there is no possibility of having two indistinguishable excitons in the same state, no ESA pathway to cancel the GSB and/or SE, and thus a signal appears on the diagonal. The inability to excite a second exciton is equivalent to having an infinite repulsive interaction between identical excitons. Hence, the presence of a signal for a two-level system is consistent with this framework of requiring interactions for peaks to appear.

It is evident from the pathways and respective peak locations shown in Supplementary Fig. 6 that the peaks which appear in the experimental data correspond to pathways that only include polarons where the excitons (either pseudospin-up or -down) are dressed by the same Fermi sea. The presence of these peaks must then be a result of interactions between

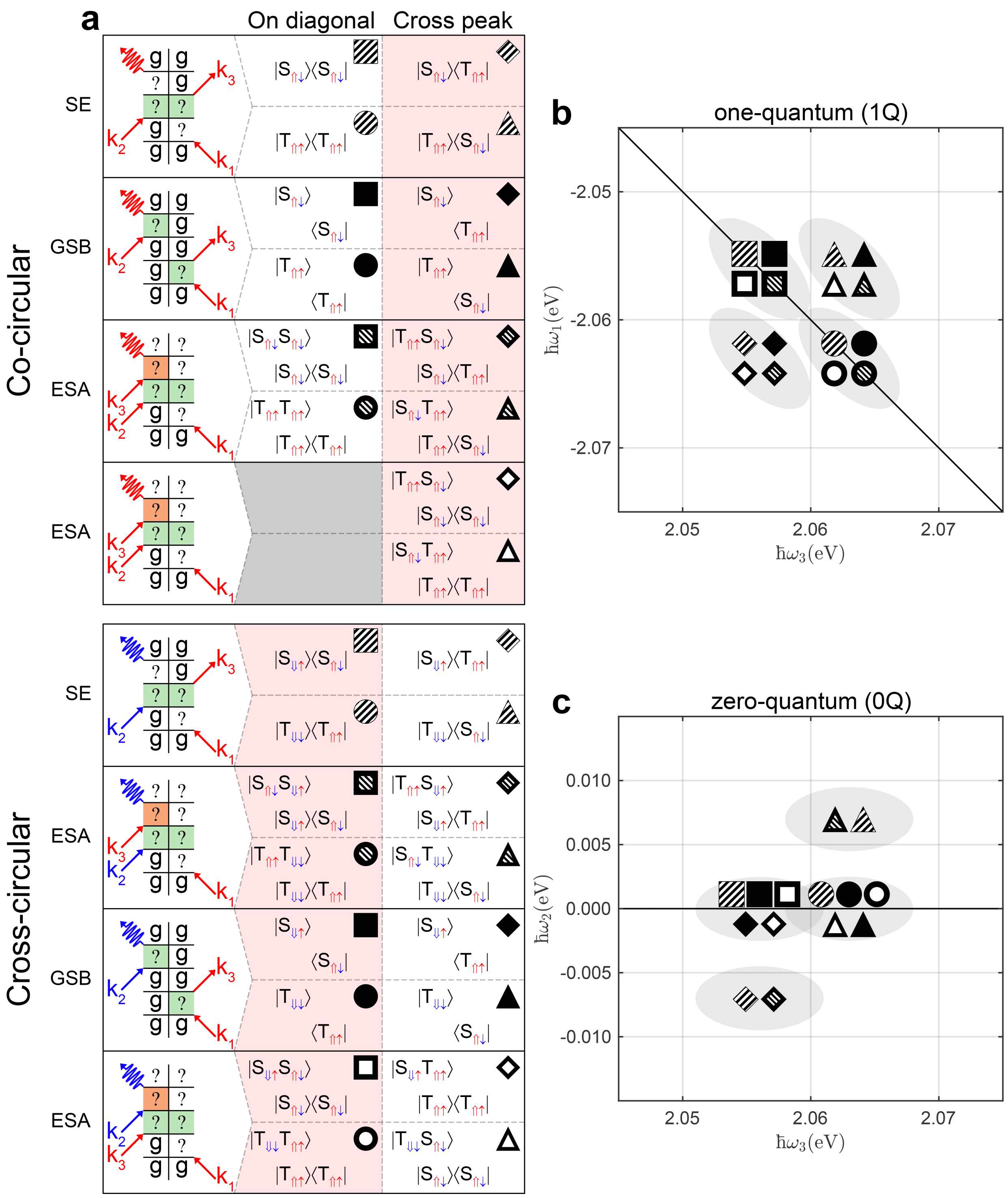

Supplementary Figure 6 | Quantum-mechanical pathway analysis. a, QM pathways for both co- and cross-circular polarizations, illustrated with Feynman-Liouville diagrams and labelled with shapes. The boxes with red backgrounds indicate pathways including states in which experimental signal is absent. The red and blue arrows indicate opposite handedness circularly polarized laser pulses, σ^+ and σ^- respectively. The listed $|\ \ \rangle$ and $\langle\ \ |$ states slot into the green (or orange for doubly excited states) boxes, to fill out the Feynman-Liouville diagram. The diagrams include the ground state (g), the attractive polaron states listed in the main manuscript, and doubly excited states. Filled shapes indicate ground state bleach (GSB), striped shapes indicate stimulated emission (SE), and hollow shapes indicate excited state absorption (ESA) pathways. The ESA pathways can include a coherent superposition during t_2, in which case the hollow section is striped the other way. The ESA pathways that include a population during t_2 are left as hollow shapes. **b**, Illustration of expected peak locations for a co-linear polarized 1Q rephasing measurement. **c**, Illustration of expected peak locations in a co-linear polarized 0Q measurement.

these polarons. In contrast, the pathways that involve polarons dressed by the two different Fermi seas (with different spin) do not produce measurable peaks in the experiments. The implication then is that interactions between polarons involving different Fermi seas are much weaker than the interactions between polarons involving the same Fermi sea.

Comparing the cross-circular 0Q spectra (Fig. 3 in main text) to the pathway analysis in this Supplementary Fig. 6 we find that the experimental peaks in the $\sigma^+\sigma^-\sigma^+\sigma^-$ and $\sigma^+\sigma^+\sigma^-\sigma^-$ also align with the pathways and diagram of Supplementary Fig. 6c. This further confirms that the QM pathways presented here are indeed the ones that contribute to the measured signals.

SUPPLEMENTARY NOTE 5: POLARON MODEL

As discussed in Methods, we describe the system using the minimal Hamiltonian

$$\hat{H}_0 = \sum_{\sigma=\Uparrow,\Downarrow} \left(E_X^0 \hat{X}_\sigma^\dagger \hat{X}_\sigma + E_T^0 \hat{T}_\sigma^\dagger \hat{T}_\sigma + E_S^0 \hat{S}_\sigma^\dagger \hat{S}_\sigma \right) + \sum_{\sigma=\Uparrow,\Downarrow} \left(\alpha_t \sqrt{N_e} \hat{T}_\sigma^\dagger \hat{X}_\sigma + \alpha_s \sqrt{N_e} \hat{S}_\sigma^\dagger \hat{X}_\sigma + \text{H.c.} \right). \tag{2}$$

Here, E_X^0, E_S^0, and E_T^0 are the exciton, singlet trion, and triplet trion energies in the absence of exciton-trion coupling, and the strength of the exciton-trion coupling at low doping is related to the Fermi energy and j trion binding energy [17]:

$$\alpha_j \sqrt{N_e} \simeq \sqrt{\frac{3}{2}} \sqrt{\varepsilon_j E_F}. \tag{3}$$

As discussed in Methods, to a good approximation the exciton couples to a coherent superposition of trions in N sites within an area A:

$$\hat{S}_\sigma = \frac{1}{\sqrt{N_e}} \sum_{j=1}^{N_e} \hat{S}_{\sigma,j}, \qquad \hat{T}_\sigma = \frac{1}{\sqrt{N_e}} \sum_{j=1}^{N_e} \hat{T}_{\sigma,j}, \tag{4}$$

This effectively leads to interactions between trions since a given electron is only available for a single trion formation, i.e.,

$$(\hat{S}_{\sigma,j})^2 = (\hat{T}_{\sigma,j})^2 = \hat{S}_{\Uparrow,j} \hat{T}_{\Downarrow,j} = \hat{T}_{\Uparrow,j} \hat{S}_{\Downarrow,j} = 0. \tag{5}$$

To proceed, we make reference to the experiment, where the signal arises from processes involving either one or two excitations. We therefore now construct an orthonormal basis for all such states. For the single excitations, this is simply

$$|X_\sigma\rangle = \hat{X}_\sigma^\dagger |0\rangle, \qquad |S_\sigma\rangle = \hat{S}_\sigma^\dagger |0\rangle, \qquad |T_\sigma\rangle = \hat{T}_\sigma^\dagger |0\rangle. \tag{6}$$

Here, the state $|0\rangle$ represents the state of the medium prior to the first pulse, which we take to consist of Fermi seas in the K and K′ valleys. For two excitations, things become more complicated since there are 21 such terms: six terms involving two identical excitations, and 15 terms involving two distinguishable excitations. Using the expansion of the trion

states in Eq. (4) together with the phase-space filling in Eq. (5), we arrive at the following orthonormal basis:

$$
\begin{aligned}
|X_\sigma, X_\sigma\rangle &= \frac{1}{\sqrt{2}} \hat{X}_\sigma^\dagger \hat{X}_\sigma^\dagger |0\rangle && \text{(7a)} \\
|T_\sigma, T_\sigma\rangle &= \frac{N_e}{\sqrt{2N_e(N_e-1)}} \hat{T}_\sigma^\dagger \hat{T}_\sigma^\dagger |0\rangle && \text{(7b)} \\
|S_\sigma, S_\sigma\rangle &= \frac{N_e}{\sqrt{2N_e(N_e-1)}} \hat{S}_\sigma^\dagger \hat{S}_\sigma^\dagger |0\rangle && \text{(7c)} \\
|S_\Uparrow, T_\Downarrow\rangle &= \frac{N_e}{\sqrt{N_e(N_e-1)}} \hat{S}_\Uparrow^\dagger \hat{T}_\Downarrow^\dagger |0\rangle && \text{(7d)} \\
|S_\Downarrow, T_\Uparrow\rangle &= \frac{N_e}{\sqrt{N_e(N_e-1)}} \hat{T}_\Uparrow^\dagger \hat{S}_\Downarrow^\dagger |0\rangle && \text{(7e)} \\
|X_\Uparrow, X_\Downarrow\rangle &= \hat{X}_\Uparrow^\dagger \hat{X}_\Downarrow^\dagger |0\rangle && \text{(7f)} \\
|X_\sigma, S_{\sigma'}\rangle &= \hat{X}_\sigma^\dagger \hat{S}_{\sigma'}^\dagger |0\rangle && \text{(7g)} \\
|X_\sigma, T_{\sigma'}\rangle &= \hat{X}_\sigma^\dagger \hat{T}_{\sigma'}^\dagger |0\rangle && \text{(7h)} \\
|S_\Uparrow, S_\Downarrow\rangle &= \hat{S}_\Uparrow^\dagger \hat{S}_\Downarrow^\dagger |0\rangle && \text{(7i)} \\
|T_\Uparrow, T_\Downarrow\rangle &= \hat{T}_\Uparrow^\dagger \hat{T}_\Downarrow^\dagger |0\rangle && \text{(7j)} \\
|S_\Uparrow, T_\Uparrow\rangle &= \hat{S}_\Uparrow^\dagger \hat{T}_\Uparrow^\dagger |0\rangle && \text{(7k)} \\
|S_\Downarrow, T_\Downarrow\rangle &= \hat{S}_\Downarrow^\dagger \hat{T}_\Downarrow^\dagger |0\rangle \,. && \text{(7l)}
\end{aligned}
$$

The normalisation of the two-exciton state in Eq. (7a) arises from the bosonic commutation relation, $[\hat{X}_\sigma^\dagger, \hat{X}_{\sigma'}] = \delta_{\sigma\sigma'}$, and the terms in Eqs. (7b) to (7e) involving blocking effects. The remaining terms involve distinguishable particles that do not have any blocking.

1. *Interactions*

While the state space in Eqs. (6) and (7) contains a large number of terms, we emphasize that this is the minimal model containing excitons in both valleys and the corresponding singlet and triplet trions, along with their possible interactions. Indeed, the interactions are effectively encoded in the normalisation of the two-excitation terms in (7b) to (7e). To see

this, consider for instance the following matrix element of the Hamiltonian

$$\langle T_\sigma, T_\sigma | \hat{H}_0 | X_\sigma, T_\sigma \rangle = \alpha_t \sqrt{2(N_e - 1)}. \tag{8}$$

The blocking effect in Eq. (5) results in the factor $\sqrt{N_e - 1}$ instead of the usual factor $\sqrt{N_e}$ arising from bosonic statistics, similar to the case of two photons coupled to atoms in a cavity [18]. The difference can therefore be viewed as an interaction due to phase-space filling. Taking all such terms into account, and expanding to leading order in $1/N_e$, we obtain the following interaction terms in the Hamiltonian,

$$\begin{aligned} \hat{H}_{int} \simeq & -\frac{1}{2\sqrt{N_e}} \sum_\sigma \left[\alpha_s \hat{S}^\dagger_\sigma \hat{S}^\dagger_\sigma \hat{X}_\sigma \hat{S}_\sigma + \alpha_t \hat{T}^\dagger_\sigma \hat{T}^\dagger_\sigma \hat{X}_\sigma \hat{T}_\sigma \right] \\ & - \frac{\alpha_s}{2\sqrt{N_e}} \left(\hat{S}^\dagger_\Uparrow \hat{T}^\dagger_\Downarrow \hat{X}_\Uparrow \hat{T}_\Downarrow + \hat{S}^\dagger_\Downarrow \hat{T}^\dagger_\Uparrow \hat{X}_\Downarrow \hat{T}_\Uparrow \right) \\ & - \frac{\alpha_t}{2\sqrt{N_e}} \left(\hat{T}^\dagger_\Uparrow \hat{S}^\dagger_\Downarrow \hat{X}_\Uparrow \hat{S}_\Downarrow + \hat{T}^\dagger_\Downarrow \hat{S}^\dagger_\Uparrow \hat{X}_\Downarrow \hat{S}_\Uparrow \right) + \text{H.c.} \end{aligned} \tag{9}$$

This explicitly demonstrates that within the model (2) we only have interactions between trions that share the same Fermi sea.

2. *Relating parameters of the model to experimental observables*

In the presence of the K and K′ Fermi seas, the frequencies of the exciton and trion peaks shift to those of the repulsive and attractive polarons. The shift of a given branch is $O(E_F)$, and at the density of the experiment we have $E_F \simeq 0.3$ meV. This is within the experimental uncertainty of any given peak position, and hence a shift of this order of magnitude is not measurable. We thus take the energies of the trions in the absence of the coupling, E^0_X, E^0_T and E^0_S, to be essentially the same as the energies observed in experiment, with a small shift to ensure that the peaks in the presence of coupling coincide with the two attractive and the one repulsive polaron peaks. This leads to the average (theory) values

$$\left\langle E^0_X \right\rangle = 2091.2 \text{ meV}, \qquad \left\langle E^0_T \right\rangle = 2063.6 \text{ meV}, \qquad \left\langle E^0_S \right\rangle = 2056.7 \text{ meV}\,, \tag{10}$$

where the average is over disorder in the sample that leads to an inhomogeneous broadening, as discussed below in Sec. A 1. We emphasize that these parameters of the model are

obtained directly from the experimental density and the frequencies of the observed peaks.

3. *Exciton valley exchange*

Finally, we remark on the possibility of including an exchange coupling between K and K′ excitons due to inter-valley electron-hole exchange. This effectively leads to exchange processes that couple $X_\Uparrow \leftrightarrow X_\Downarrow$ as well as $S_{\Uparrow\downarrow} \leftrightarrow T_{\Downarrow\downarrow}$ and $S_{\Downarrow\uparrow} \leftrightarrow T_{\Uparrow\uparrow}$. This process is expected to scale linearly with momentum at low exciton momenta, and for the momentum associated with the pulses in our experiment, we estimate the rate to be $\sim 20\,\mu$eV [19]. This is much smaller than any other relevant energy scale, and therefore the exchange process can be ignored for the excitons.

The rate of exciton exchange inside a trion is more involved, since the excitons now move relative to the electron. In this case, the rate of such exchange has previously been measured in $MoSe_2$ to be ~ 2.9 meV [20]. We have checked that including a trion exchange coupling of this magnitude in our MDCS spectra does not lead to any visible difference compared with the spectra in the absence of exchange; thus, in all results presented in the manuscript we have taken the exchange to zero in order to reduce the number of theoretical parameters.

A. MDCS Simulation details

We now discuss how the MDCS is simulated within the model (2). We will do this using two approaches. The first is based on correlation functions, and this has been used to calculate all results presented in the main text, as discussed in the Methods. The second approach is based on a master equation description of the system. As we demonstrate, these two approaches yield comparable results.

1. *Approach based on correlation functions*

As discussed in Methods, the third order response contributing to the rephasing experimental protocols illustrated in Supplementary Fig. 3 is due to ground state bleach, stimulated emission, and excited state absorption [21]. Their contributions to the material polarization

are

$$
\begin{aligned}
P^{(3)}_{\sigma\sigma'} = &-\left(\frac{i}{\hbar}\right)^3 \mu_X^4 E_0^3 \Big(\mathrm{Tr}\left[\hat{\rho}_0 \hat{X}_{\sigma'} e^{i\hat{H}_0 t_1} \hat{X}^\dagger_\sigma e^{i\hat{H}_0(t_2+t_3)} \hat{X}_\sigma e^{-i\hat{H}_0 t_3} \hat{X}^\dagger_{\sigma'}\right] \\
&\qquad + \mathrm{Tr}\left[\hat{\rho}_0 \hat{X}_{\sigma'} e^{i\hat{H}_0(t_1+t_2)} \hat{X}^\dagger_{\sigma'} e^{i\hat{H}_0 t_3} \hat{X}_\sigma e^{-i\hat{H}_0(t_2+t_3)} \hat{X}^\dagger_\sigma\right] \\
&\qquad - \mathrm{Tr}\left[\hat{\rho}_0 \hat{X}_{\sigma'} e^{i\hat{H}_0(t_1+t_2+t_3)} \hat{X}_\sigma e^{-i\hat{H}_0 t_3} \hat{X}^\dagger_{\sigma'} e^{-i\hat{H}_0 t_2} \hat{X}^\dagger_\sigma\right] \Big) \\
= &-\left(\frac{i}{\hbar}\right)^3 \mu_X^4 E_0^3 \Big(\mathrm{Tr}\left[\hat{\rho}_0 \hat{X}_{\sigma'} e^{i\hat{H}_0 t_1} \hat{X}^\dagger_\sigma\right] \mathrm{Tr}\left[\hat{\rho}_0 \hat{X}_\sigma e^{-i\hat{H}_0 t_3} \hat{X}^\dagger_{\sigma'}\right] \\
&\qquad + \mathrm{Tr}\left[\hat{\rho}_0 \hat{X}_{\sigma'} e^{i\hat{H}_0(t_1+t_2)} \hat{X}^\dagger_{\sigma'}\right] \mathrm{Tr}\left[\hat{\rho}_0 \hat{X}_\sigma e^{-i\hat{H}_0(t_2+t_3)} \hat{X}^\dagger_\sigma\right] \\
&\qquad - \mathrm{Tr}\left[\hat{\rho}_0 \hat{X}_{\sigma'} e^{i\hat{H}_0(t_1+t_2+t_3)} \hat{X}_\sigma e^{-i\hat{H}_0 t_3} \hat{X}^\dagger_{\sigma'} e^{-i\hat{H}_0 t_2} \hat{X}^\dagger_\sigma\right] \Big). \qquad (11)
\end{aligned}
$$

The trace is over the possible states of the system, with the density matrix $\hat{\rho}_0 = |0\rangle\langle 0|$ describing the initial state of the system prior to any excitations.

The exact time evolution of the contributions in Eq. (11) using the Hamiltonian in Eq. (2) can be straightforwardly calculated since the expectation value is taken over a state that corresponds to the vacuum of excitons and trions. However, to obtain the spectra, in practice it is simplest to first Fourier transform to frequency space. For instance, in the case of single quantum spectroscopy, this Fourier transform is with respect to t_1 and t_3 at fixed t_2. Including a homogeneous linewidth Γ, for the ground state bleach term we have

$$
\begin{aligned}
&\int_0^\infty dt_1\, dt_3\, e^{i(\omega_1+i\Gamma)t_1} e^{i(\omega_3+i\Gamma)t_3} \mathrm{Tr}\left[\hat{\rho}_0 \hat{X}_{\sigma'} e^{i\hat{H}_0 t_1} \hat{X}^\dagger_\sigma\right] \mathrm{Tr}\left[\hat{\rho}_0 \hat{X}_\sigma e^{-i\hat{H}_0 t_3} \hat{X}^\dagger_{\sigma'}\right] \\
&\qquad = -\mathrm{Tr}\left[\hat{\rho}_0 \hat{X}_{\sigma'} \frac{1}{\omega_1 + \hat{H}_0 + i\Gamma} \hat{X}^\dagger_\sigma\right] \mathrm{Tr}\left[\hat{\rho}_0 \hat{X}_\sigma \frac{1}{\omega_3 - \hat{H}_0 + i\Gamma} \hat{X}^\dagger_{\sigma'}\right]. \qquad (12)
\end{aligned}
$$

The other terms are Fourier transformed similarly. Note that some care must be taken in the two-exciton terms, since t_3 can appear in multiple time-evolution operators.

Apart from the homogeneous broadening described by Γ, we also have inhomogeneous broadening. We model this as dominated by dielectric disorder due to spatial fluctuations of the substrate and the presence of impurities and adsorbates, in accordance with the results of Ref. [22]. This leads to fluctuations of the attractive and repulsive polaron energies, leading to an approximately Gaussian lineshape (see Sec. A 2) of width σ_A and σ_R, respectively. Importantly, the fluctuations in the energies are correlated [22], and since $\sigma_A \simeq \sigma_R$ we

simply average the signal by varying the input parameters E_X^0, E_S^0 and E_T^0 around their average positions in Eq. (10) according to

$$\left\langle P^{(3)}_{\sigma\sigma'} \right\rangle_{\text{disorder}} = \int d\epsilon \frac{e^{-\epsilon^2/2\sigma_R^2}}{\sqrt{2\pi}\sigma_R} P^{(3)}_{\sigma\sigma'} \left(E_X^0 = \left\langle E_X^0 \right\rangle + \epsilon, E_S^0 = \left\langle E_S^0 \right\rangle + \epsilon \frac{\sigma_A}{\sigma_R}, E_T^0 = \left\langle E_T^0 \right\rangle + \epsilon \frac{\sigma_A}{\sigma_R} \right). \tag{13}$$

2. Master equation description

An alternative approach is to model the MDCS response via a master equation description, as is more common in the literature. This approach has the advantage of including decoherence processes more generally, rather than simply via a phenomenological dephasing parameter as given above. However for the 28-level system considered, it also has considerable drawbacks. One is simply the additional computational time required to perform the simulations in the large state space of the density matrix of the system, as well as including the effects of inhomogeneous broadening. Another more subtle issue is that there is not enough information to define all the decoherence channels unambigously. The effects of various dephasing and relaxation channels all contribute to the MDCS spectra in different ways, but can not easily be distinguished without a large number of additional measurements. To address these issues, we perform key simulations of interest with both approaches, *without* inhomogeneous broadening. This allows us to confirm that our theoretical approach is valid without over-fitting to the experimental results.

To simulate the MDCS spectra using a master equation approach, we implement a similar approach to that detailed in Ref. [23, 24]. The master equation is assumed to be of Lindblad type [25–27]

$$\dot{\rho} = -\frac{i}{\hbar}[\hat{H}_0, \rho] + \sum_j \gamma_j \left(\hat{L}_j \rho \hat{L}_j^\dagger - \frac{1}{2} \left\{ \hat{L}_j^\dagger \hat{L}_j, \rho \right\} \right) \tag{14}$$

where γ_j and $\hat{L}_j$ represent the decoherence rates and operators respectively.

We solve the evolution of the master equation in tetradic representation or 'super-operator' form [23, 28]. This involves expressing the density matrix in vector form, making the evaluation of the time evolution simpler, at the expense of larger memory requirements.

The Lindblad equation therefore takes the form

$$\dot{\vec{\rho}}(t) = \mathcal{L}\vec{\rho}(t) \tag{15}$$

where

$$\mathcal{L} = -i\left(\hat{H}_0 \otimes I - I \otimes \hat{H}_0^T\right) + \sum_j \gamma_j \left(\hat{L}_j \otimes \hat{L}_j^* - \frac{1}{2}\left\{\hat{L}_j^\dagger \hat{L}_j \otimes I + I \otimes (\hat{L}_j^\dagger \hat{L}_j)^T\right\}\right) \tag{16}$$

where $\otimes$ indicates the Kronecker product and I is the identity matrix with the same dimensions as the Hamiltonian. The time evolution of the density matrix can then be written in terms of the propagator $G(t) = e^{\mathcal{L}t}$,

$$\vec{\rho}(t) = G(t)\vec{\rho}(0) \tag{17}$$

where the propagator can be Fourier transformed analytically [23]

$$G(\omega) = \frac{1}{i\omega - \mathcal{L}} \tag{18}$$

to give the frequency response.

We can now define the MDCS response in terms of the following expressions

$$P^{(3)}(t_1, t_2, t_3) \propto -\sum_j R_j \tag{19}$$

$$R_1 = \left\langle \hat{\mu}^- G(t_3)\hat{V}^+ \rho_0 \hat{V}^- G(t_1)\hat{V}^+ G(t_2)\right\rangle, \tag{20}$$

$$R_2 = -\left\langle \hat{\mu}^- G(t_3)\hat{V}^+ G(t_2)\hat{V}^+ \rho_0 \hat{V}^- G(t_1)\right\rangle, \tag{21}$$

$$R_3 = \left\langle \hat{\mu}^- G(t_2)\hat{V}^+ \rho_0 \hat{V}^- G(t_1)\hat{V}^+ G(t_3)\right\rangle, \tag{22}$$

$$R_4 = \left\langle \hat{\mu}^- G(t_3)\hat{V}^- G(t_2)\hat{V}^+ G(t_1)\hat{V}^+ \rho_0\right\rangle, \tag{23}$$

$$R_5 = -\left\langle \hat{\mu}^- G(t_3)\hat{V}^+ G(t_1)\hat{V}^+ \rho_0 \hat{V}^- G(t_2)\right\rangle, \tag{24}$$

$$R_6 = \left\langle \hat{\mu}^- G(t_1)\hat{V}^+ \rho_0 \hat{V}^- G(t_2)\hat{V}^+ G(t_3)\right\rangle, \tag{25}$$

$$R_7 = \left\langle \hat{\mu}^- G(t_3)\hat{V}^+ G(t_2)\hat{V}^- G(t_1)\hat{V}^+ \rho_0\right\rangle, \tag{26}$$

$$R_8 = -\left\langle \hat{\mu}^- G(t_2)\hat{V}^+ G(t_1)\hat{V}^+ \rho_0 \hat{V}^- G(t_3)\right\rangle. \tag{27}$$

where ρ_0 is the initial (steady state) density matrix of the system. These response functions R_j can also be used to define the rephasing (R_1, R_2, R_3), non-rephasing (R_5, R_6, R_7) and double-quantum (R_4, R_8) contributions to the MDCS spectra. In the comparison that follows, we only compute and compare to the rephasing spectrum ($\sum_{j=1}^{3} R_j$). In the expression above we assume that the dipole operator for each transition consists of Pauli x-operators, such that $\hat{V} = -\hat{\mu} \cdot \vec{E} = -\sigma^x \left(\vec{\mu} \cdot \vec{E}\right) = \hat{V}^+ + \hat{V}^-$.

The system Hamiltonian, as described above and in the main text can be expressed in terms of matrix elements as follows. Note that the interactions (i.e., blocking effects) are encoded in the basis states and thus we use $\hat{H}_0$. The single particle states are,

$$\langle X_\sigma | \hat{H}_0 | X_\sigma \rangle = E_X^0 \tag{28a}$$

$$\langle S_\sigma | \hat{H}_0 | S_\sigma \rangle = E_S^0 \tag{28b}$$

$$\langle T_\sigma | \hat{H}_0 | T_\sigma \rangle = E_T^0 \tag{28c}$$

where $\sigma = \{\Uparrow, \Downarrow\}$ which correspond to the K and K′ valleys respectively.

The diagonal terms are,

$$\langle X_\sigma, X_{\sigma'} | \hat{H}_0 | X_\sigma, X_{\sigma'} \rangle = 2E_X^0 \tag{29a}$$

$$\langle X_\sigma, S_{\sigma'} | \hat{H}_0 | X_\sigma, S_{\sigma'} \rangle = E_X^0 + E_S^0 \tag{29b}$$

$$\langle X_\sigma, T_{\sigma'} | \hat{H}_0 | X_\sigma, T_{\sigma'} \rangle = E_X^0 + E_T^0 \tag{29c}$$

$$\langle S_\sigma, S_{\sigma'} | \hat{H}_0 | S_\sigma, S_{\sigma'} \rangle = 2E_S^0 \tag{29d}$$

$$\langle T_\sigma, T_{\sigma'} | \hat{H}_0 | T_\sigma, T_{\sigma'} \rangle = 2E_T^0 \tag{29e}$$

$$\langle S_\sigma, T_{\sigma'} | \hat{H}_0 | S_\sigma, T_{\sigma'} \rangle = E_S^0 + E_T^0. \tag{29f}$$

where both $\sigma = \{\Uparrow, \Downarrow\}$ and $\sigma' = \{\Uparrow, \Downarrow\}$ in the respective K-valleys but avoiding double counting of states when the state is indistinguishable on swapping valleys.

The cross terms can be slightly more complex. Specifically, we have

$$\langle X_\sigma, S_{\sigma'}| \hat{H}_0 |X_\sigma, X_{\sigma'}\rangle = \alpha_s\sqrt{N_e(1+\delta_{\sigma,\sigma'})} \tag{30a}$$

$$\langle X_\sigma, T_{\sigma'}| \hat{H}_0 |X_\sigma, X_{\sigma'}\rangle = \alpha_t\sqrt{N_e(1+\delta_{\sigma,\sigma'})} \tag{30b}$$

$$\langle S_\sigma, S_\sigma| \hat{H}_0 |X_\sigma, S_\sigma\rangle = \alpha_s\sqrt{2(N_e-1)} \tag{30c}$$

$$\langle T_\sigma, T_\sigma| \hat{H}_0 |X_\sigma, T_\sigma\rangle = \alpha_t\sqrt{2(N_e-1)} \tag{30d}$$

$$\langle S_\sigma, T_\sigma| \hat{H}_0 |X_\sigma, S_\sigma\rangle = \alpha_t\sqrt{N_e} \tag{30e}$$

$$\langle S_\sigma, T_\sigma| \hat{H}_0 |X_\sigma, T_\sigma\rangle = \alpha_s\sqrt{N_e} \tag{30f}$$

$$\langle S_\Uparrow, T_\Downarrow| \hat{H}_0 |X_\Uparrow, T_\Downarrow\rangle = \alpha_s\sqrt{N_e-1} \tag{30g}$$

$$\langle S_\Downarrow, T_\Uparrow| \hat{H}_0 |X_\Downarrow, T_\Uparrow\rangle = \alpha_s\sqrt{N_e-1} \tag{30h}$$

$$\langle S_\Downarrow, T_\Uparrow| \hat{H}_0 |X_\Uparrow, S_\Downarrow\rangle = \alpha_t\sqrt{N_e-1} \tag{30i}$$

$$\langle S_\Uparrow, T_\Downarrow| \hat{H}_0 |X_\Downarrow, S_\Uparrow\rangle = \alpha_t\sqrt{N_e-1} \tag{30j}$$

$$\langle S_\Downarrow, S_\Uparrow| \hat{H}_0 |X_\Uparrow, S_\Downarrow\rangle = \alpha_s\sqrt{N_e} \tag{30k}$$

$$\langle S_\Uparrow, S_\Downarrow| \hat{H}_0 |X_\Downarrow, S_\Uparrow\rangle = \alpha_s\sqrt{N_e} \tag{30l}$$

$$\langle T_\Uparrow, T_\Downarrow| \hat{H}_0 |X_\Uparrow, T_\Downarrow\rangle = \alpha_t\sqrt{N_e} \tag{30m}$$

$$\langle T_\Downarrow, T_\Uparrow| \hat{H}_0 |X_\Downarrow, T_\Uparrow\rangle = \alpha_t\sqrt{N_e} \tag{30n}$$

In this basis the dipole operators are the creation and annihilation operators for the exciton states in the two valleys, ie. $\hat{V}^+ = \hat{X}_\sigma^\dagger$. As the indistinguishable states are not duplicated in the state space, the corresponding creation/annihilation operators pick up an additional factor of $\sqrt{2}$. This is required to ensure the correct cancellation of the signal when there are no interactions between states.

To mimic the phenomenological dephasing implemented in the correlation function approach above, we include the following Lindblad operators,

$$\hat{L}_{X_\sigma} = \hat{X}_\sigma^\dagger \hat{X}_\sigma \tag{31a}$$

$$\hat{L}_{S_\sigma} = \hat{S}_\sigma^\dagger \hat{S}_\sigma \tag{31b}$$

$$\hat{L}_{T_\sigma} = \hat{T}_\sigma^\dagger \hat{T}_\sigma \tag{31c}$$

Each operator is then included with the same dephasing rate Γ discussed in the previous

section. In principle a number of other effects could be included via additional Lindblad terms, including exciton recombination and correlated dephasing within and between valleys. However these effects are considerably more difficult to extract reliably from the MDCS data. We therefore limit the comparison to the pure dephasing discussed above.

In Supplementary Fig. 7 we show the simulated 1Q rephasing spectra with co- and cross-circular polarization, computed via the two approaches. For accurate comparison, these plots do not include the effects of inhomogeneous broadening. We see very good correspondence between the approaches. It is worth noting the minor differences in peak width and magnitudes which stem from the different approach to including dephasing in the calculations.

It is also worth noting that the amplitude of the exciton peaks, and the exciton-trion cross-peaks compared to the trion peaks are relatively larger in the simulations than in the experimental measurements. This can be mostly clearly seen in Fig. 2f of the main text, at the edges of the observed energy window. It is likely that these discrepancies also stem from the idealised decoherence model used, as loss processes and additional dephasing types can reduce this signal. However more detailed measurements and theoretical analysis are required to understand the details.

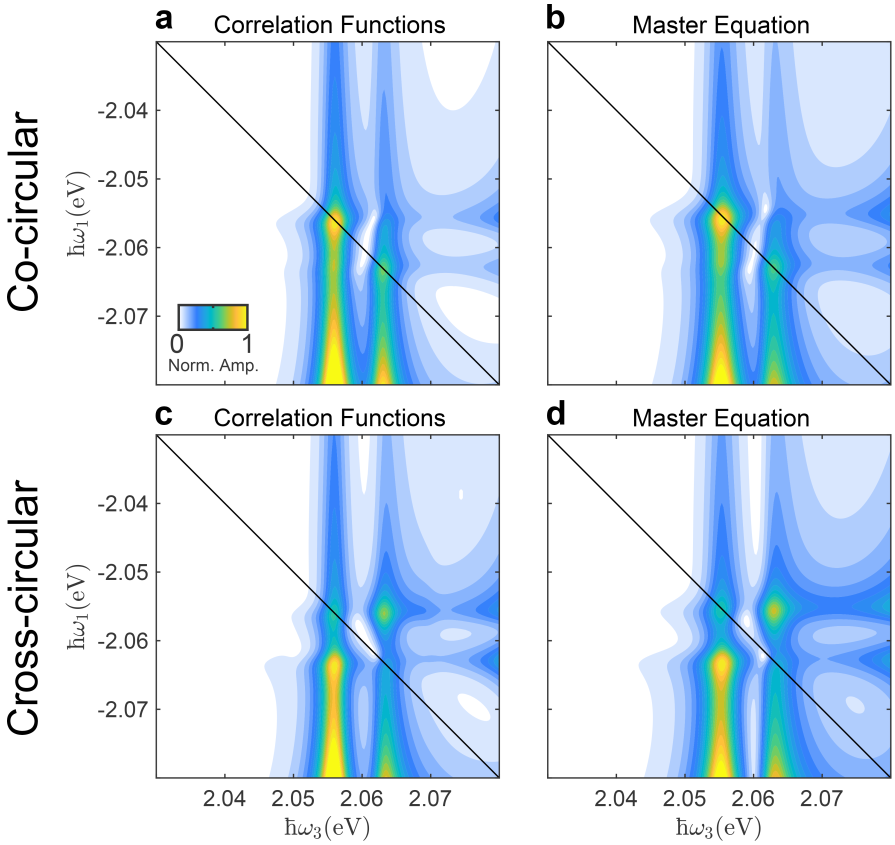

Supplementary Figure 7 | Comparison of simulation models. a-d, Simulated 1Q rephasing 2D spectra with co-circular polarization using correlator code (a) and master equation code (b). Cross-polarized scheme using the correlator (c) and master equation code (d). To make both models easier to compare, no inhomogeneous broadening is included here. Both models run with fitted experimental values from Supplementary Fig. 13, and estimated electron/exciton densities.

SUPPLEMENTARY NOTE 6: BIPOLARON STATES IN WS_2

In this section we provide more evidence and detailed explanation behind the attribution of the bipolaron peaks in the 1Q 2D spectrum (Fig. 2d) discussed in the main text. To aid this discussion, Supplementary Fig. 8 shows the energies of and separations between all of the singly excited states and unbound doubly excited states. Also shown are the biexciton and bipolaron states.

Confirmation that these peaks come from doubly excited states

The negative peaks in the real part of the 1Q 2D spectrum (Supplementary Fig. 4a of the main text), give a strong indication that these peaks arise from excited state absorption pathways, and thus involve doubly excited states. However, there are other possible effects that can generate negative peaks, such as excitation induced shifts [29] or bandgap renor-

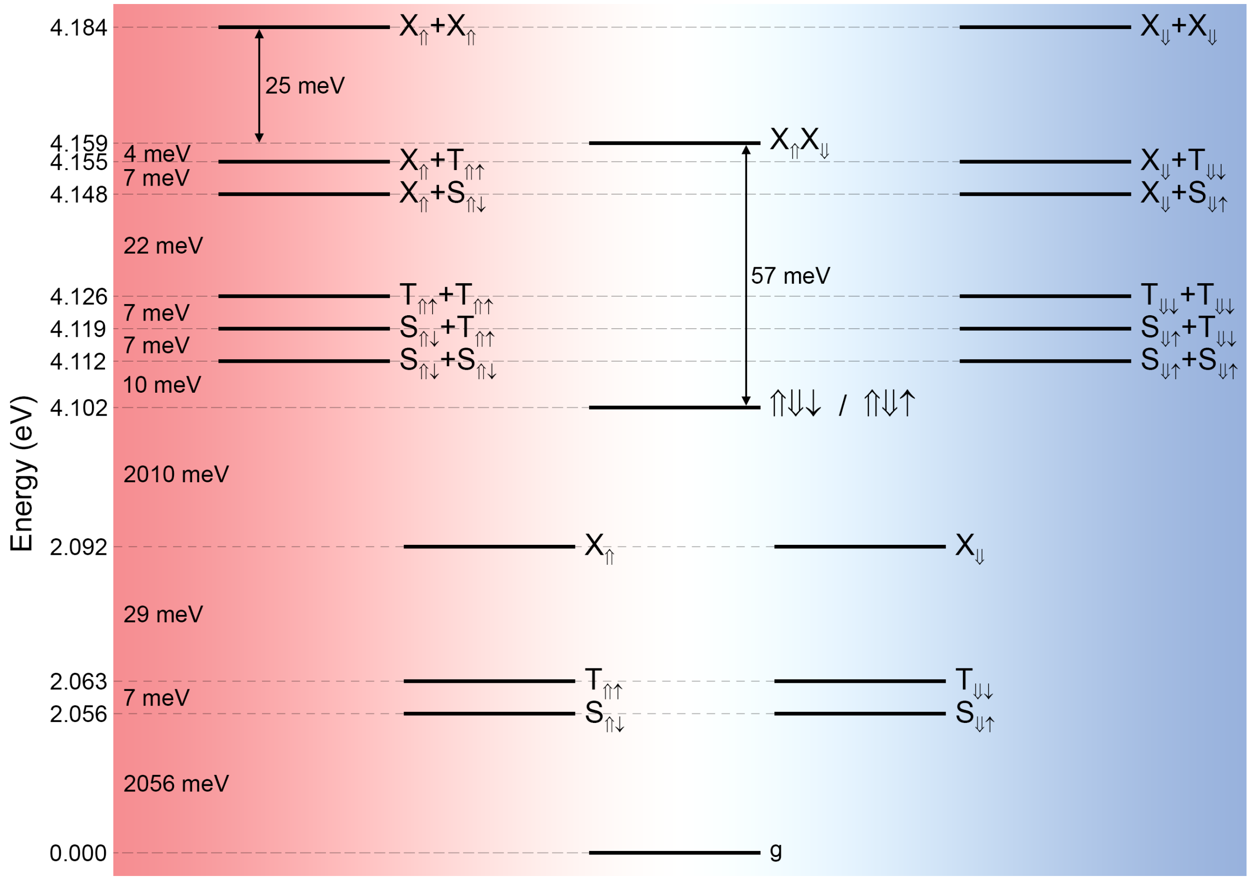

Supplementary Figure 8 | Energy level diagram for possible excited states in WS_2. Energies of the repulsive and attractive polaron states and complexes are indicated, with the exciton component as pseudospin-up (red shading) or pseudospin-down (blue shading). The stated biexciton binding energy is as from [15].

malisation. One unambiguous means to identify doubly excited states is two-quantum (2Q) MDCS [15, 30]. In this type of measurement the first two pulses create a coherent superposition between the ground state and any doubly excited states (a 2Q coherence). The third pulse can then drive the system into a (1Q) coherent superposition of singly and doubly excited states or ground and singly excited states. These types of pathways are depicted in Supplementary Fig. 9, along with the expected locations of the corresponding peaks in a 2Q 2D spectrum. The 2D spectrum correlates $\hbar\omega_{2Q}$, the energy of the 2Q coherence, with the emission energy, $\hbar\omega_3$. Thus, peaks a 2Q 2D spectrum provide unambiguous confirmation of the presence and energy of doubly excited states.

Supplementary Figure 9 shows the experimental 2Q spectrum, possible QM pathways, and their respective peak locations. The fact that a broad peak occurs in the 2Q spectrum at $\hbar\omega_{2Q} = 4.105 \pm 0.005\,\text{eV}$, $\hbar\omega_3 = 2.052 \pm 0.050\,\text{eV}$ in Supplementary Fig. 9n, shows that doubly excited states are present and contribute to the third-order signal. With reference to

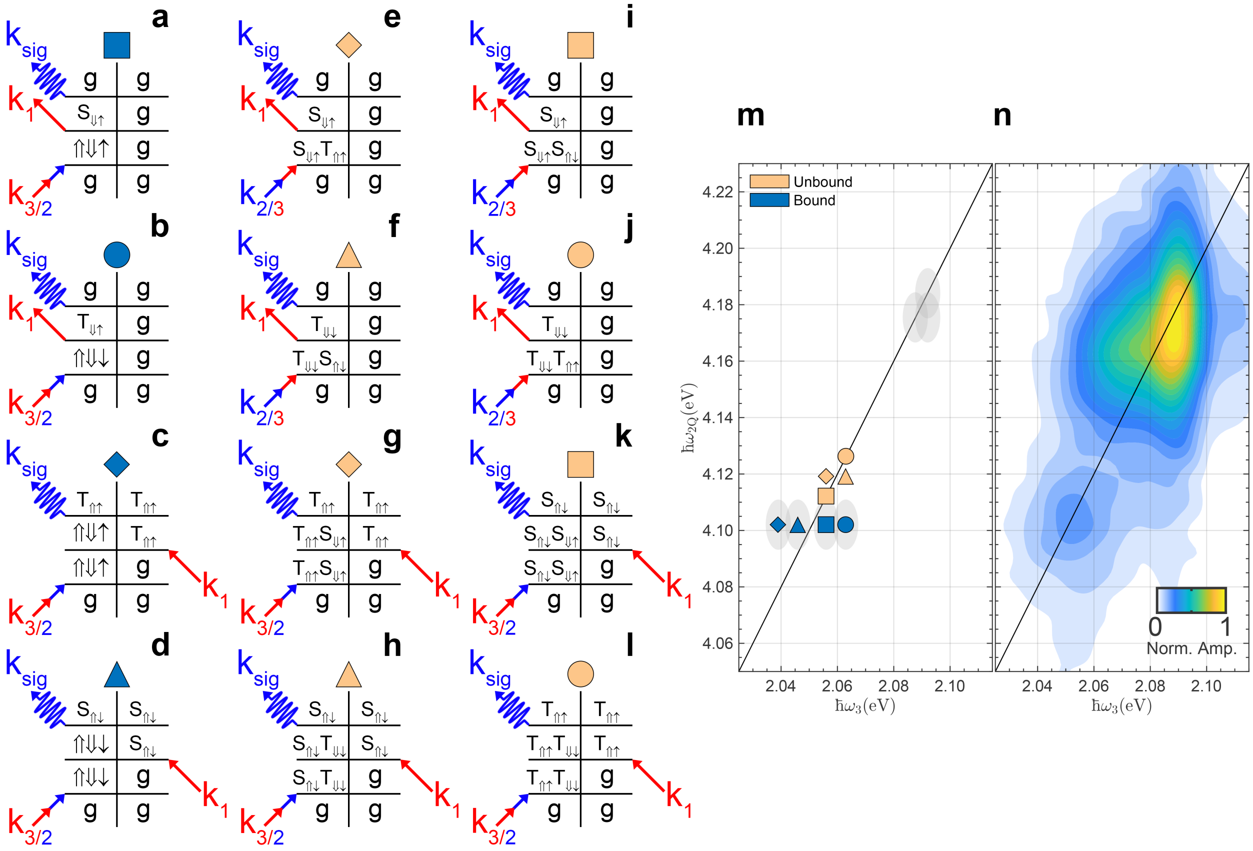

Supplementary Figure 9 | Bipolaron 2Q pathway analysis. a-l, 2Q pathways for excitation of the bipolaron (⇑⇓↑ and ⇑⇓↓). In this case, the $\mathbf{k}_2$ and $\mathbf{k}_3$ pulses arrive simultaneously so the t_1 Feynman diagram rung has been removed for simplicity. **m**, illustration of peak locations in a cross-circular 2Q spectrum arising from pathways in (a-l). **n**, Experimental cross-circular 2Q spectrum.

the energy level diagram in Supplementary Fig. 8, the location of the peak clearly matches what would be expected for the bipolaron, based on the energies from the 1Q measurements, that is: $\hbar\omega_{2Q} = 4.102\,\text{eV}$, with $\hbar\omega_3$ peaks expected at 2.039, 2.046, 2.056, and 2.063 eV.

In these 2Q measurements there is no rephasing, so inhomogeneous broadening blurs the peaks, making it impossible to resolve the different peaks. However, it is clear that there is a broad peak observed around these energies, which provides strong confirmation that the negative peaks in the 1Q 2D spectrum arise from excited state absorption pathways involving these doubly excited states. It is possible that the unbound S + S, S + T, and T + T doubly excited states identified in Supplementary Fig. 8 could contribute to this broad peak via the pathways in Supplementary Fig. 9e-l. However the $\hbar\omega_{2Q}$ energies expected are 10, 17, and 24 meV above the bipolaron energy, and all well above the centre of the peak in the experimental results. Hence even if they do contribute, they cannot explain the whole signal.

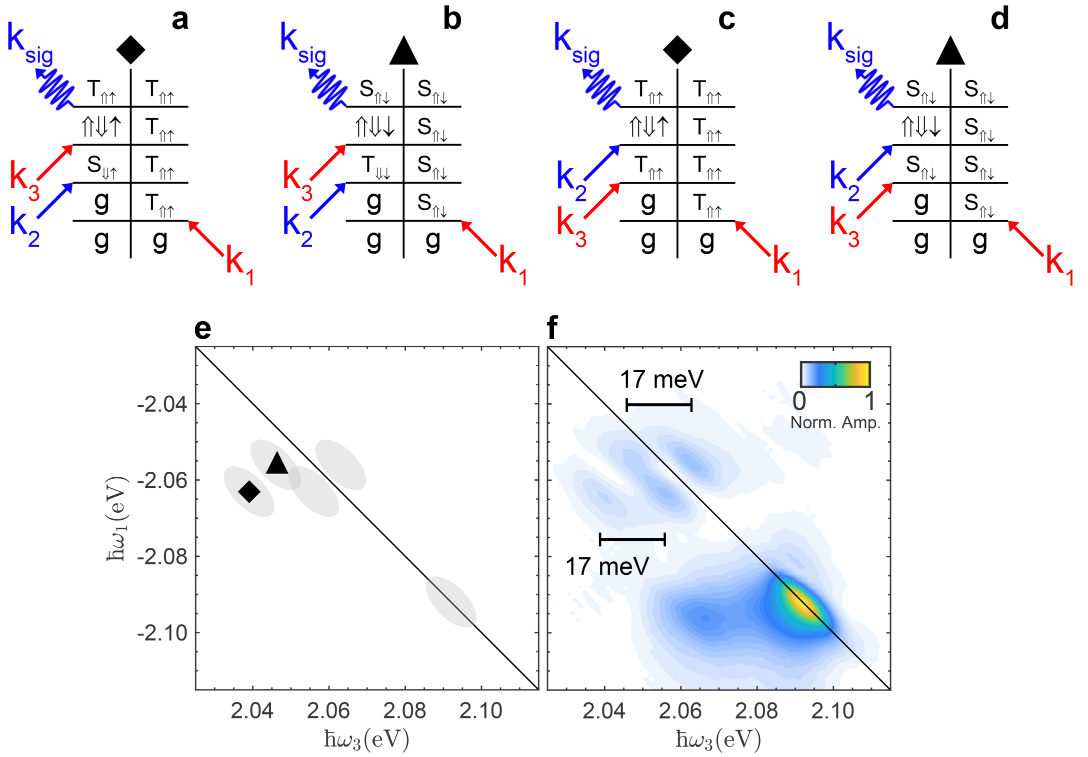

Supplementary Figure 10 | Bipolaron 1Q pathway analysis. a-d, 1Q pathways for excitation of the bipolaron. **e**, Illustration of peak locations in a cross-circular 1Q spectrum arising from pathways in (a-d). **f**, Experimental cross-circular 1Q spectrum.

Pathways leading to the peaks arising from the bipolaron states

The pathways that contribute to the bipolaron peaks in the 1Q 2D spectra are shown in Supplementary Fig. 10. Pathways for both the $\sigma^+\sigma^-\sigma^+\sigma^-$ and $\sigma^+\sigma^+\sigma^-\sigma^-$ are shown, since the results showed that both contribute to these peaks. For each, there are two possible pathways with $\hbar\omega_1 = \hbar\omega_S$ and $\hbar\omega_1 = \hbar\omega_T$. The emission energy for these pathways can be determined by taking the difference between the energy of the bipolaron $\Uparrow\Downarrow\downarrow$ (or equivalently the $\Uparrow\Downarrow\uparrow$) state and the final state of the system, as can be seen in Fig. 4c in the main text. From the location of the peaks, we determine the energy of the bipolaron states to be 4.102 eV. This gives emission energies 17 meV below the S and T energies.

If we then consider the binding energy to be the minimum energy required to break apart this complex, then the lowest energy combination it could be split into would be X + S (as per the energy level diagram in Supplementary Fig. 8), all other pairs of the included quasiparticles (X + T or XX + e) are higher in energy. This gives a binding energy of 46 ± 3 meV, which is significantly higher than predicted when considering a charged biexciton as two-body exciton + trion bound state. This large binding energy is indicative

of the cooperative binding between all three quasiparticles.

Another key to identifying these bipolarons as arising from cooperatively bound charged biexcitons comes from the observation of pathways in Supplementary Fig. 10a,b, that on the left hand side of the diagrams there is initial absorption to the $S_{\Downarrow\uparrow}$ ($T_{\Downarrow\downarrow}$) followed by excitation to the bipolaron $\Uparrow\Downarrow\uparrow$ ($\Uparrow\Downarrow\downarrow$). The emission from the bipolaron then leaves behind a $T_{\Uparrow\uparrow}$ ($S_{\Uparrow\downarrow}$) polaron. That is, the $\uparrow$ ($\downarrow$) electrons that initially dress the $\Downarrow$ exciton are left dressing the $\Uparrow$ exciton after going via the bipolaron. This process requires that in the bipolaron state both excitons are interacting strongly with the electron.

Alternative pathways that could lead to these peaks and why we rule them out

In the process of identifying the pathways and nature of the bipolaron peaks, we considered alternate pathways and possible bound states, but ultimately ruled them all out. Below we detail pathways for alternate bound states and how they deviate from our experimental observations.

Supplementary Figure 11 considers the pathways for the case of a simple two-body binding of an exciton to a trion as the basis for these peaks. For each of these a different binding energy is required for XS (46 meV) and XT (53 meV), which is not so unreasonable. However, in Supplementary Fig. 11(a-d), the pathways that yield the observed peaks in the 1Q 2D spectrum, the system evolves over t_2 as a superposition between X and S (or X and T) states, which should show up as a peak at $\omega_2 = 36\,\text{meV}$ (or 29 meV) in the 0Q 2D spectrum for the $\sigma^+\sigma^-\sigma^+\sigma^-$ pulse ordering. No such peaks are observed, the only peaks in the 0Q spectrum emitting at 2.039 - 2.046 eV occur at $\pm 7\,\text{meV}$, consistent with the bipolaron pathways identified in Supplementary Fig. 10.

In principle the pathways in Supplementary Fig. 11e,f could generate the peaks in the 1Q 2D spectrum and are consistent with the peaks at $\hbar\omega_2 = 0$ in the 0Q spectrum. However, in this case we would also expect peaks arising from the pathways in (g-h), which would appear at $\hbar\omega_1$ equal to the exciton energy and $\hbar\omega_3 = 2.01\,\text{eV}$, and no such peak is observed. Similarly, peaks from (c-d) should also contribute to this position, yet no peaks are observed. We therefore rule out the possibility of a two-body bound exciton-trion state as contributing to these bipolaron peaks.

Another possibility is that these peaks arise from a doubly charged biexciton, consisting

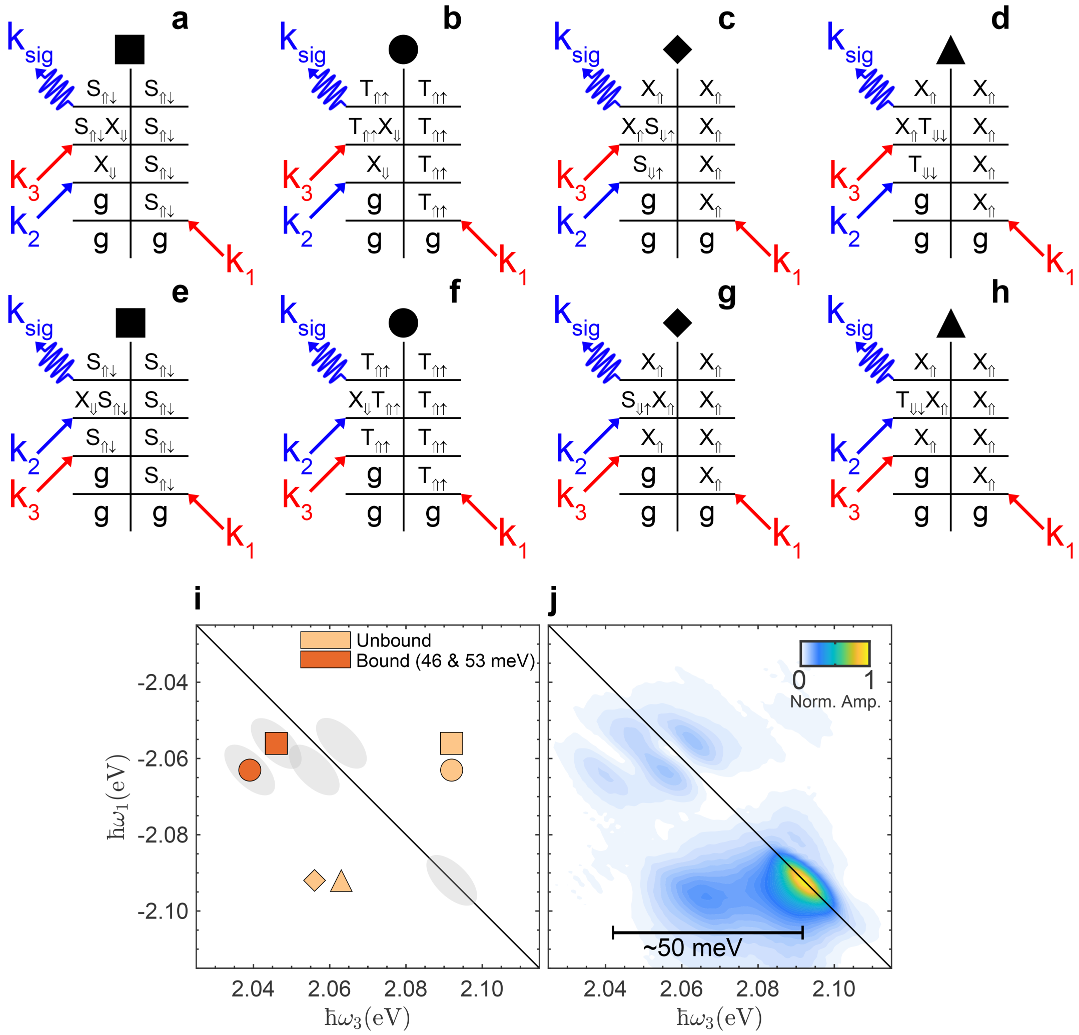

Supplementary Figure 11 | Bound exciton-trion 1Q pathway analysis. a-h, Possible QM pathways for exciton-trion states. **i**, illustration of peak locations in a cross-circular 1Q spectrum arising from pathways in (a-h). **j**, Experimental cross-circular 1Q spectrum for reference.

of two trions bound together. Possible pathways for these states are shown in Supplementary Fig. 12. Just considering these pathways, the peaks seen in the 1Q spectrum could arise from the pathways (a-d) involving a T + S bound state with a binding energy of 17 meV, being the energy by which the peaks are shifted to lower $\hbar\omega_3$ values. However, these pathways suggest bound states between trions with electrons in the same band (i.e. $T_{\Uparrow\Uparrow} + S_{\Downarrow\Uparrow}$ or $S_{\Uparrow\Downarrow} + T_{\Downarrow\Downarrow}$). It is expected that Pauli blocking would prevent the formation of such a bound state involving indistinguishable electrons. The other possible bound states in Supplementary Fig. 12(e-h) involve four electrons and two holes that are all distinguishable. For these square and circle pathways to generate peaks in Supplementary Fig. 12j at the triangle and diamond locations, there would need to be a different binding energy for the S + S (10 meV) and T +

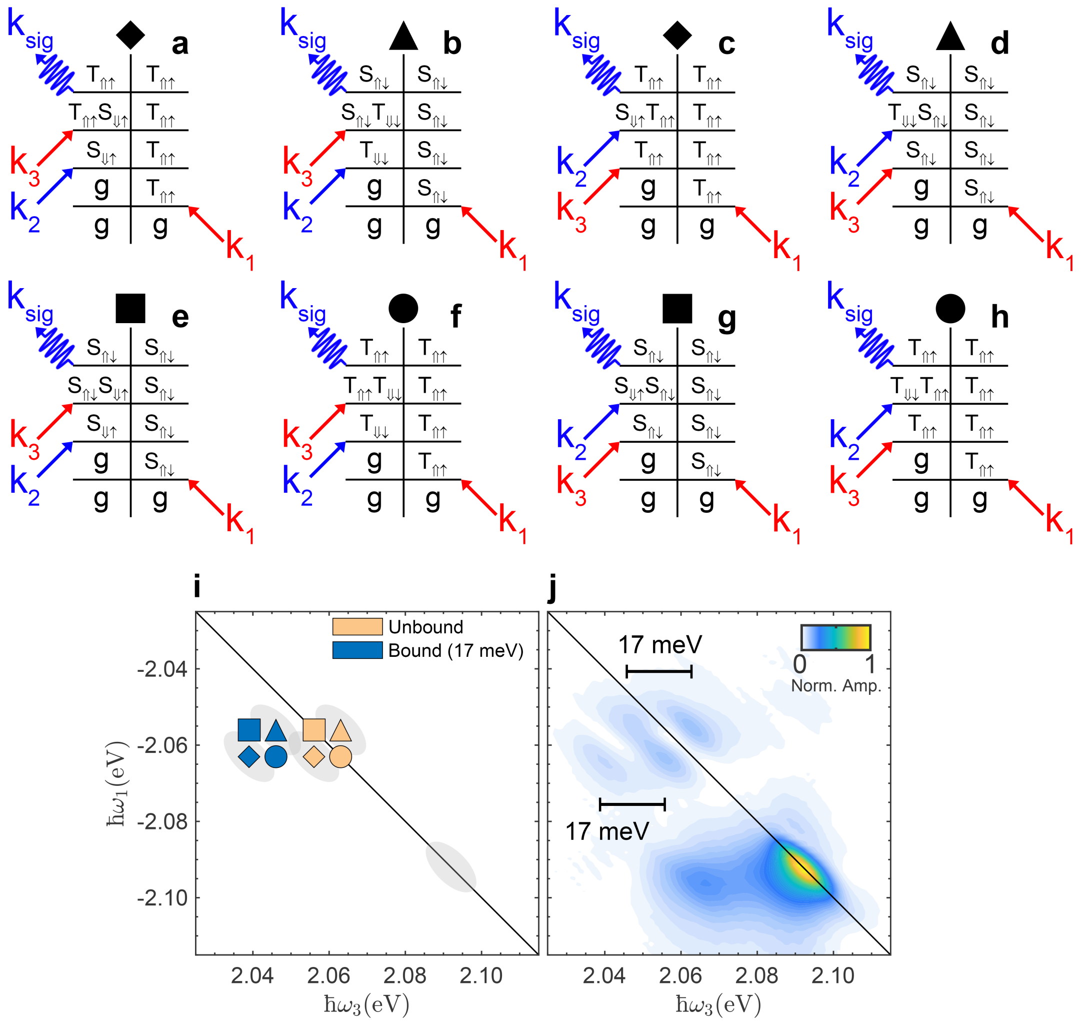

Supplementary Figure 12 | Doubly charged biexciton 1Q pathway analysis. a-h, 1Q excited state absorption (ESA) pathways. **i** illustration of peak locations in a cross-circular 1Q spectrum arising from pathways in (a-h). Red crossed shapes indicate QM pathways in which the attractive polarons are bound to different Fermi seas. **j** Experimental cross-circular 1Q spectrum for reference.

T (24 meV) bound states, which is unlikely, given they both involve the same combination of electrons and holes. Furthermore, Monte Carlo calculations indicate that the 6-body bound state is not stable [31]. Thus, we rule out the possibility that these pathways, and the associated doubly charged biexciton state, contribute to the signal measured.

Further comparison of the data and states identified here with previous PL data

Photoluminescence (PL) measurements on WS_2 and WSe_2 have also identified a peak attributed to a charged biexciton, emitting ~ 50 meV below the exciton energy [32–36]. The timescales in photoluminescence measurements are intrinsically different, as they are time

averaged measurements, and the optically excited states typically have time to relax to the lower energy dark exciton states, or in doped systems to dark trion states. For the case of a dark trion, the exciton and electron cannot be in the same valley as this would mean the electrons are in the same band, hence only the inter-valley configuration is allowed. It is thus assumed that in PL measurements the charged biexciton consists of a dark exciton in one valley, a bright exciton the other valley and an electron in the same valley as the bright exciton. The emission is then either from the bright exciton, leaving behind a dark trion; or from the bright trion, leaving behind a dark exciton. In PL measurements there is no way to distinguish between these two possibilities, and different authors have made the case for both, which leads to vastly different binding energies being determined. In all cases it appears to be assumed, even if not explicitly stated, that the binding is between an exciton and a trion. However, the MDCS measurements presented here indicate that the charged biexciton is formed as a result of the strong interaction between each of the two excitons and electron. This picture of a cooperatively bound charged biexciton is also consistent with PL measurements, the only difference being that because one of the excitons is a dark exciton, the emission will always be from the bright exciton and leave behind a dark, inter-valley trion. That is, while in the MDCS measurements we see emission at two different $\hbar\omega_3$ values, the PL will show only one spectral peak. We therefore conclude that the PL measurements that report this charged biexciton should consider the emission as leaving behind a trion, and determine the binding energy based on the spectral shift from the exciton emission.

SUPPLEMENTARY NOTE 7: FITS TO 1Q & 0Q 2D SPECTRA

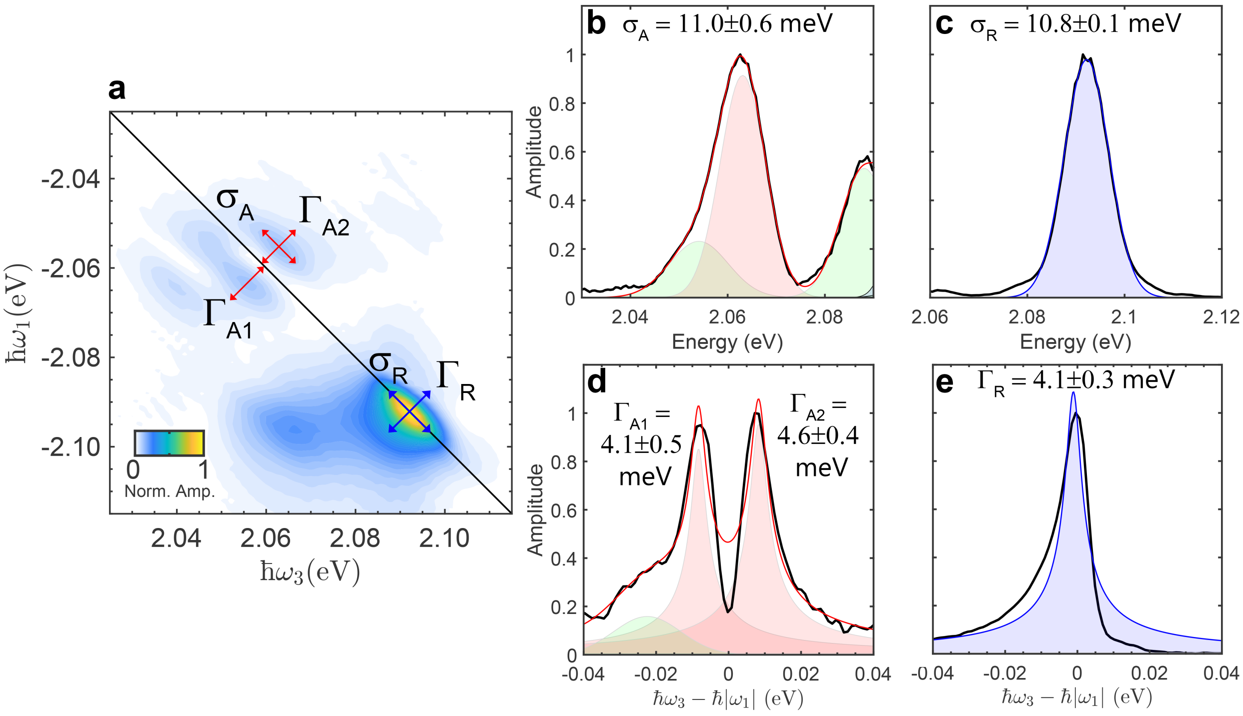

Supplementary Figure 13 | One-quantum linewidth fits. **a**, Experimental cross-circularly polarized 1Q 2D amplitude spectrum. Red (blue) arrows indicate slices at the attractive (repulsive) polaron peak for diagonal and anti-diagonal. **b-c**, Diagonal slices (black) of (a) along the σ line for both the attractive (b) and repulsive polaron (c), fit with Gaussian profiles (red or blue respectively). Peaks appearing to the sides of the attractive polaron (shaded green), are due to the edges of other peaks. The inhomogeneous linewidth (σ) is shown above for Gaussian FWHM in meV. **d-e**, Anti-diagonal slices (black) of (a) along the Γ arrows, for both attractive (d) and repulsive polaron (e). The linewidth is fit with a square-root-Lorentzian, with FWHM in meV displayed above the fits. In (d) there are two peaks from the two singlet-triplet cross-peaks. Uncertainties are derived from the 95% confidence bounds of the fits.

Further details of interactions between quasiparticles and their local environment can be obtained from the shape and width of the 2D peaks in MDCS plots. Here we provide these numbers as a step towards further analysis and understanding of these interactions. We fit anti-diagonal and diagonal slices of the 2D spectral amplitudes with square-root-Lorentzian (Lorentzian for intensity) and Gaussian models respectively, assuming we are in the inhomogeneous limit [37]. In this limit, for diagonal peaks the width along the diagonal is the inhomogeneous linewidth, and along the anti-diagonal the width corresponds to the homogeneous linewidth. For cross-peaks with correlated inhomogeneous broadening the anti-diagonal width can be a measure of the strength of the correlation but is also related to the homogeneous linewidths of the two transitions. In Supplementary Fig. 13, the diagonal and anti-diagonal linewidths of the attractive and repulsive polaron are similar.

Supplementary Figure 14 shows 0Q 2D spectra of the two cross-circular schemes ($\sigma^+\sigma^+\sigma^-\sigma^-$

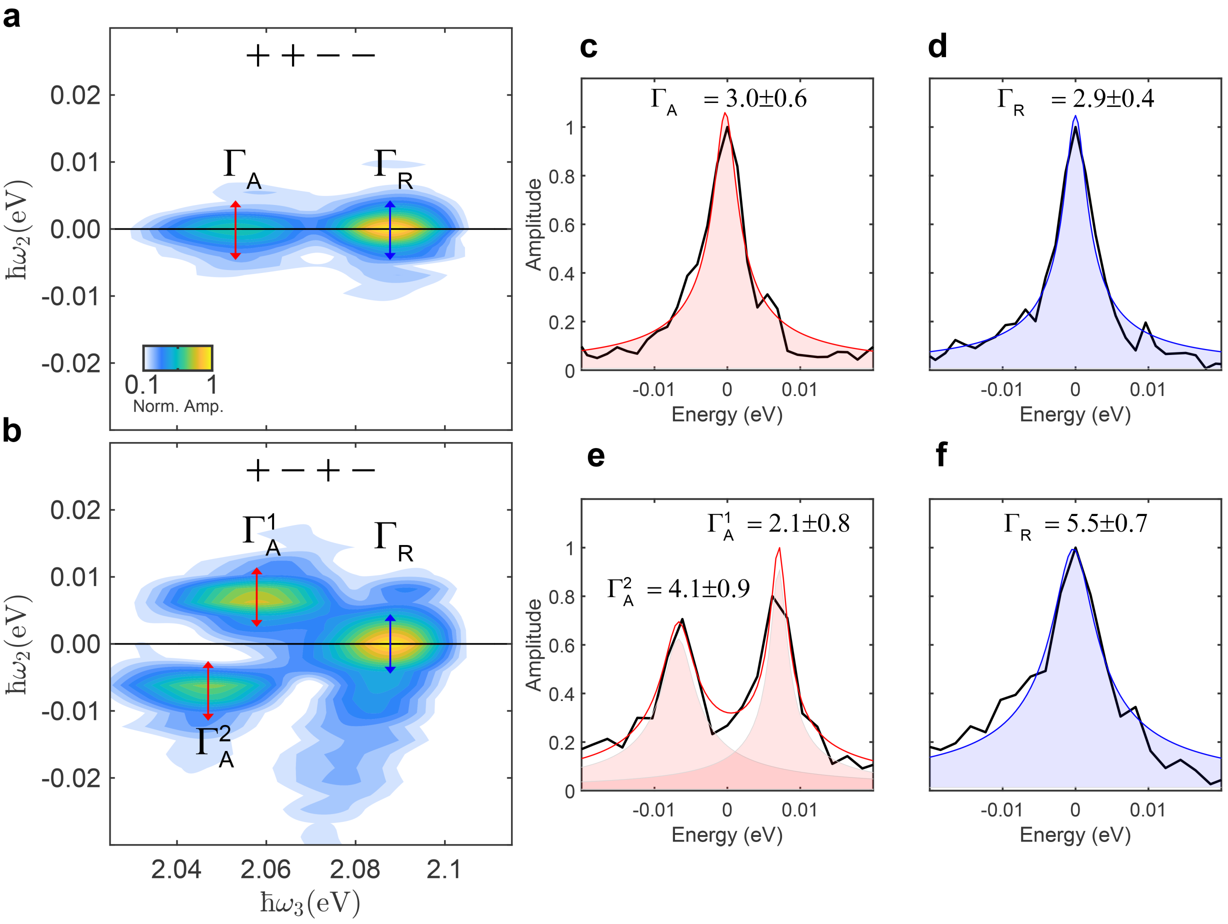

Supplementary Figure 14 | Zero-quantum linewidth fits. a-b, Experimental $\sigma^+\sigma^+\sigma^-\sigma^-$ (a) and $\sigma^+\sigma^-\sigma^+\sigma^-$ 0Q 2D spectra. Vertical arrows indicate the point at which slices were taken for (c-f). The peaks in (a) are from QM pathways that evolve via a population state, where the linewidth along $\hbar\omega_2$ is related to the population lifetime. The peaks in (b) evolve via coherent superpositions between excitations in different valleys (e.g. $|T_{\Uparrow\uparrow}\rangle\langle S_{\Downarrow\uparrow}|$, $|S_{\Uparrow\downarrow}\rangle\langle T_{\Downarrow\downarrow}|$). In this case the linewidth along $\hbar\omega_2$ gives an upper limit for the decoherence rate of these inter-valley coherences. **c-f**, Vertical slices of the attractive (red) and repulsive (blue) polarons for both (a) and (b), fit with square-root-Lorentzian profile where Γ (FWHM) is shown in meV.

and $\sigma^+\sigma^-\sigma^+\sigma^-$) with vertical slices through the peaks. For the $\sigma^+\sigma^-\sigma^+\sigma^-$ polarization sequence, where the system evolves as a coherence between excitations in the K and K$'$ valley (e.g. $|T_{\Uparrow\uparrow}\rangle\langle S_{\Downarrow\uparrow}|$ or $|S_{\Uparrow\downarrow}\rangle\langle T_{\Downarrow\downarrow}|$), the linewidths are related to the decoherence time of these inter-valley coherences: $T_2 = \frac{2\hbar}{\Gamma}$, where Γ is the FWHM of the Lorentzian fit in meV. For the $\sigma^+\sigma^+\sigma^-\sigma^-$ polarization scheme the system evolves via population states and the widths are related to the state lifetimes. In this case however, they are likely limited by the constraints of our experiment.

REFERENCES

1. Earl, S. K. *et al.* Coherent dynamics of Floquet-Bloch states in monolayer WS_2 reveals fast adiabatic switching. *Phys. Rev. B* **104,** L060303 (2021).

2. Zhang, H. *et al.* Interference effect on optical signals of monolayer MoS_2. *Appl. Phys. Lett.* **107,** 101904 (2015).

3. Zhumagulov, Y. V., Vagov, A., Gulevich, D. R., Junior, P. E. F. & Perebeinos, V. Trion induced photoluminescence of a doped MoS2 monolayer. *J. Chem. Phys.* **153,** 044132 (2020).

4. Robert, C. *et al.* Spin/valley pumping of resident electrons in WSe_2 and WS_2 monolayers. *Nat. Commun.* **12,** 5455 (2021).

5. Shang, J. *et al.* Observation of Excitonic Fine Structure in a 2D Transition-Metal Dichalcogenide Semiconductor. *ACS Nano* **9,** 647–655 (2015).

6. Xu, W. *et al.* Determining the Optimized Interlayer Separation Distance in Vertical Stacked 2D WS_2:hBN:MoS_2 Heterostructures for Exciton Energy Transfer. *Small* **14,** 1703727 (2018).

7. Chow, P. K. *et al.* Defect-Induced Photoluminescence in Monolayer Semiconducting Transition Metal Dichalcogenides. *ACS Nano* **9,** 1520–1527 (2015).

8. Mak, K. F. *et al.* Tightly bound trions in monolayer MoS_2. *Nat. Mater.* **12,** 207–211 (2012).

9. Sebait, R., Biswas, C., Song, B., Seo, C. & Lee, Y. H. Identifying Defect-Induced Trion in Monolayer WS_2 via Carrier Screening Engineering. *ACS Nano* **15,** 2849–2857 (2021).

10. Tongay, S. *et al.* Broad-Range Modulation of Light Emission in Two-Dimensional Semiconductors by Molecular Physisorption Gating. *Nano Lett.* **13,** 2831–2836 (2013).

11. Robertson, J. High dielectric constant oxides. *EPJ Appl. Phys.* **28,** 265–291 (2004).

12. Sidler, M. *et al.* Fermi polaron-polaritons in charge-tunable atomically thin semiconductors. *Nat. Phys.* **13,** 255–261 (2016).

13. Kormányos, A. *et al.* k·p theory for two-dimensional transition metal dichalcogenide semiconductors. *2D Mater.* **2,** 022001 (2015).

14. Jakubczyk, T. *et al.* Radiatively Limited Dephasing and Exciton Dynamics in $MoSe_2$ Monolayers Revealed with Four-Wave Mixing Microscopy. *Nano Lett.* **16,** 5333–5339 (2016).

15. Conway, M. A. *et al.* Direct measurement of biexcitons in monolayer WS_2. *2D Mater.* **9,** 021001 (2022).

16. Mukamel, S. Multidimensional Femtosecond Correlation Spectroscopies of Electronic and Vibrational Excitations. *Annu Rev Phys Chem* **51,** 691–729 (2000).

17. Efimkin, D. K., Laird, E. K., Levinsen, J., Parish, M. M. & MacDonald, A. H. Electron-exciton interactions in the exciton-polaron problem. *Phys. Rev. B* **103,** 075417 (2021).

18. Quantum effects of the atom-cavity interaction on four-wave mixing. *Opt. Commun.* **62,** 328–332 (1987).

19. Yu, T. & Wu, M. W. Valley depolarization due to intervalley and intravalley electron-hole exchange interactions in monolayer MoS_2. *Phys. Rev. B* **89,** 205303 (2014).

20. Hao, K. *et al.* Trion valley coherence in monolayer semiconductors. *2D Mater.* **4,** 025105 (2017).

21. Hamm, P. Principles of nonlinear optical spectroscopy: A practical approach or: Mukamel for dummies. *University of Zurich* **41,** 77 (2005).

22. Raja, A. *et al.* Dielectric disorder in two-dimensional materials. *Nat. Nanotechnol.* **14,** 832–837 (2019).

23. Plenio, M. B., Almeida, J. & Huelga, S. F. Origin of long-lived oscillations in 2D-spectra of a quantum vibronic model: Electronic versus vibrational coherence. *J. Chem. Phys.* **139,** 235102 (2013).

24. Lim, J. *et al.* Signatures of spatially correlated noise and non-secular effects in two-dimensional electronic spectroscopy. *J. Chem. Phys.* **146,** 024109 (2017).

25. Manzano, D. A short introduction to the Lindblad master equation. *AIP Adv.* **10,** 025106 (2020).

26. Gorini, V. Completely positive dynamical semigroups of N-level systems. *J Math Phys* **17,** 821 (1976).

27. Lindblad, G. On the generators of quantum dynamical semigroups. *Commun. Math. Phys.* **48,** 119–130 (1976).

28. Knap, M., Arrigoni, E., von der Linden, W. & Cole, J. H. Emission characteristics of laser-driven dissipative coupled-cavity systems. *Phys. Rev. A* **83,** 023821 (2011).

29. Singh, R. *et al.* Polarization-dependent exciton linewidth in semiconductor quantum wells: A consequence of bosonic nature of excitons. *Phys. Rev. B* **94,** 081304 (2016).

30. Karaiskaj, D. *et al.* Two-Quantum Many-Body Coherences in Two-Dimensional Fourier-Transform Spectra of Exciton Resonances in Semiconductor Quantum Wells. *Phys. Rev. Lett.* **104,** 117401 (2010).

31. Mostaani, E. *et al.* Diffusion quantum Monte Carlo study of excitonic complexes in two-dimensional transition-metal dichalcogenides. *Phys. Rev. B* **96,** 075431 (2017).

32. Paur, M. *et al.* Electroluminescence from multi-particle exciton complexes in transition metal dichalcogenide semiconductors. *Nat. Commun.* **10,** 1709 (2019).

33. Chatterjee, S. *et al.* Probing biexciton in monolayer WS_2 through controlled many-body interaction. *2D Mater.* **9,** 015023 (2021).

34. Chen, S.-Y., Goldstein, T., Taniguchi, T., Watanabe, K. & Yan, J. Coulomb-bound four- and five-particle intervalley states in an atomically-thin semiconductor. *Nat. Commun.* **9,** 3717 (2018).

35. Barbone, M. *et al.* Charge-tuneable biexciton complexes in monolayer WSe_2. *Nat. Commun.* **9,** 3721 (2018).

36. Ye, Z. *et al.* Efficient generation of neutral and charged biexcitons in encapsulated WSe_2 monolayers. *Nat. Commun.* **9,** 3718 (2018).

37. Siemens, M. E., Moody, G., Li, H., Bristow, A. D. & Cundiff, S. T. Resonance lineshapes in two-dimensional Fourier transform spectroscopy. *Opt. Express* **18,** 17699–17708 (2010).
